# Thwarting Metabolic Dysfunction-Associated Fatty Liver Disease (MAFLD) with Common Bean: Dose- and Sex-Dependent Protection against Hepatic Steatosis

**DOI:** 10.3390/nu15030526

**Published:** 2023-01-19

**Authors:** Tymofiy Lutsiv, John N. McGinley, Elizabeth S. Neil, Michelle T. Foster, Henry J. Thompson

**Affiliations:** 1 Cancer Prevention Laboratory, Colorado State University, Fort Collins, CO 80523, USA; 2 Department of Food Science and Human Nutrition, Colorado State University, Fort Collins, CO 80523, USA

**Keywords:** hepatic steatosis, fatty liver disease, non-alcoholic fatty liver disease, metabolic dysfunction-associated fatty liver disease, common bean, pulses, lipid metabolism, biological sex

## Abstract

Hepatic steatosis signifies onset of metabolic dysfunction-associated fatty liver disease (MAFLD) caused by disrupted metabolic homeostasis compromising liver function. Regular consumption of common beans reduces the risk of metabolic impairment, but its effective dose, the impact of biological sex, and underlying mechanisms of action are unknown. We fed female and male C57BL6/J mice with obesogenic yet isocaloric diets containing 0%, 17.5%, 35%, and 70% of total dietary protein derived from cooked whole common beans. Liver tissue was collected for histopathology, lipid quantification, and RNA-seq analyses. Beans qualitatively and quantitatively diminished hepatic fat deposition at the 35% dose in female and 70% dose in male mice. Bean-induced differentially expressed genes (DEGs) most significantly mapped to hepatic steatosis and revealed dose-responsive inhibition of *de novo* lipogenesis markers (*Acly*, *Acaca*, *Fasn*, *Elovl6*, *Scd1*, etc.) and triacylglycerol biosynthesis, activation of triacylglycerol degradation, and downregulation of sterol regulatory element-binding transcription factor 1 (SREBF1) signaling. Upregulated fatty acid β-oxidation was more prominent in females, while suppression of *Cd36*-mediated fatty acid uptake—in males. Sex-dependent bean effects also involved DEGs patterns downstream of peroxisome proliferator-activated receptor α (PPARα) and MLX-interacting protein-like (MLXIPL). Therefore, biological sex determines amount of common bean in the diet required to prevent hepatic lipid accumulation.

## 1. Introduction

The 21st century is distinguished by globalization of sedentary lifestyle (owing to mechanization, automation, and computerization of labor), dietary patterns (especially the unbalanced Western-type), and the consequential upsurge in the burden of non-communicable diseases (such as obesity, type II diabetes mellitus, cardiovascular diseases, and cancer) which account for more than 70% of deaths per annum globally [1,2,3]. Despite their multifactorial etiology, disrupting the organism’s metabolic homeostasis is fundamental to their pathogenesis [3,4]. The liver plays a central role in regulating whole-body metabolism in mammals, and the dietary-induced chronic liver disorder is referred to as metabolic dysfunction-associated fatty liver disease, or MAFLD—formerly known in the biomedical literature as “non-alcoholic fatty liver disease” (NAFLD). In 2020, an international group of experts from 22 countries reached a consensus to adopt the new terminology—MAFLD—thereby emphasizing dysregulation of metabolism as the leading pathophysiological mechanism of the disease [5,6,7]. This terminology will be used henceforth.

As it progresses, MAFLD encompasses a broad continuum of chronic and heterogenous liver comorbidities. Phenotypically, hepatic steatosis [8]—the abnormal intrahepatic retention of lipids in the cytoplasm of hepatocytes—is observed. Approximately 1 in 5 individuals with hepatic steatosis progress to ectopic lipid deposition with concurrent hepatocyte damage, inflammation, and early-stage fibrosis, which signifies metabolic dysfunction-associated steatohepatitis (MASH) [9]. In turn, 20–25% of people with MASH within ten years develop advanced fibrosis with severely impaired liver function termed cirrhosis, the end-stage liver disease, markedly increasing the probability of hepatocellular carcinoma (HCC) development [5,9]. By 2030, MAFLD-induced HCC will be the leading reason for liver transplantation, exceeding hepatitis C [10,11]. Today, the overall prevalence of MAFLD worldwide is 32.4% (with a 95% confidence interval of [29.9%, 34.9%]) [12]. Around 70–90% of overweight or obese individuals as well as people with type II diabetes also suffer from MAFLD and exhibit dyslipidemia, lipotoxicity, insulin resistance, and hypertension as features of MAFLD [8,13]. Therefore, to tackle such a complex systemic challenge such as MAFLD, there is an urgent need for effective preventative strategies, such as adopting food patterns to improve whole-body health status.

Pulses are dry seeds of grain legumes unique in their content of small molecules, dietary fiber, and protein and their negligible content of lipids [14]. Owing to their profile of nutrients and bioactive compounds, pulses can be termed *superfoods*. However, their regular consumption has practically disappeared from the dietary patterns of developed countries and beyond—a change that has occurred concomitantly with the global increase in chronic disease burden. As discussed in [15], incorporating pulses into the diet can improve the quality of food patterns, and population data have reported reduced occurrences of diseases associated with metabolic dysfunction in high pulse consumers [16,17,18]. These reports are consistent with our findings that pulse consumption has an anti-obesogenic activity in rodent models of polygenic and dietary-induced obesity, including effects on liver lipid metabolism [19,20,21,22,23,24]. However, the minimal pulse dose required to elicit discernible effects on the hepatic phenotype, the impact of biological sex, and underlying molecular mechanisms have not been extensively elucidated. 

Therefore, we focused on the most consumed type of pulse—common bean, *Phaseolus vulgaris* L., to assess its effects on the development of high fat diet induced MAFLD. The objectives of this study were: (1) to quantify hepatic lipid content and determine the liver histopathological response to increasing dose of dietary bean consumption; (2) to characterize the impact of the biological sex on the bean-induced outcomes; and (3) to analyze the liver transcriptomic signature to deduce candidate molecular mechanisms underlying observed bean-induced phenotypes.

## 2. Materials and Methods

### 2.1. Animal Feeding Study

C57BL6/J female and male mice (stock #000664) were obtained from the Jackson Laboratory (Barr Harbor, ME, USA) at twenty days of age. Standard husbandry conditions were applied: polycarbonate rodent cages with a solid bottom, *ad libitum* access to distilled water and food, a 12-h light/dark cycle, 27.5 ± 2 °C room temperature. Adaptation to the housing routine and a purified diet containing 32.5% kcal fat based on the formulation D12266B (Research Diets Inc., New Brunswick, NJ, USA), as described in [21], continued until eight weeks of age, after which 20 animals were assigned by staggered randomization by body weight to each experimental diet group and fed their respective experimental diet formulations for 12–14 weeks (see Section 2.2). Animals were euthanized by cervical dislocation following isoflurane-induced anesthesia. Liver was excised, snap-frozen in liquid nitrogen, and samples stored at −70 °C until evaluated. All the procedures performed on animals complied with the Colorado State University Institutional Animal Care and Use Committee guidelines (protocol KP 1431).

### 2.2. Diet Formulations

Experimental diets were formulated so as to remain equal in total dietary energy content enabling evaluation of the direct effects from the bean. All diets were composed of equal macronutrient proportions weight by weight, i.e., 58.6% total carbohydrate, 15.5% total fat, 20% total protein, and 5.9% micronutrients, according to the AIN-93G recommendations. Mice received 32.5% kcal derived from fat, which is within the range for both total lipid dietary practice in humans [25] and simultaneously for obesogenic studies using the C57BL6/J animal model [26]. Diet containing 11% dietary fat energy was used as a low-fat and bean-free control. All diets were comprised of purified ingredients, with the exception of bean. White kidney beans were cooked as a whole food, processed with leachate, freeze-dried, and then homogenized into a fine powder. Bean doses were calculated as 0% (bean-free control), 17.5%, 35%, and 70% of total dietary protein derived from bean. Total dietary fiber concentration of bean was 23.5 g/100 g bean powder [27]. The formulation of the experimental diets is shown in Supplemental Table S1.

### 2.3. Histopathology

A small portion of frozen liver from each animal (*n* = 2 per group) was embedded in Tissue-Tek OCT compound (Sakura Finetek, Torrance, CA, USA) on a cryostat chuck and quickly frozen in the cryostat (Leica Biosystems, Deer Park, IL, USA) using a heat sink. Frozen sections of liver were cut at 10 µm, mounted on Histobond slides (Statlab Medical Products, McKinney, TX, USA), placed in pre-cooled plastic slide boxes, and stored at −70 °C until ready for staining.

#### 2.3.1. Fixation

Frozen section slides were removed from the −70 °C freezer, one stain set at a time, placed in a vertical slide rack and allowed to thaw at room temperature for 5 min prior to immersion in fixative, 10% neutral buffered formalin for 5 min.

#### 2.3.2. Hematoxylin and Eosin (H&E) Staining

Formalin-fixed slides were rinsed in deionized water, stained with Harris hematoxylin for 2 min, rinsed in deionized water, and counterstained in eosin Y for 1 min. Slides were dehydrated in a series of graded ethanols, starting with two changes of 95% ethanol, two changes of 100% ethanol, and one change of isopropanol, then cleared in four changes of xylene before mounting with MM24 mounting media (Leica Biosystems, Deer Park, IL, USA) and glass coverslips.

#### 2.3.3. Trichrome Staining

Formalin-fixed slides were rinsed in deionized water and stained using the Gomori one step aniline blue trichrome stain kit (Newcomer Supply, Middleton, WI, USA). Briefly, slides were immersed in Bouin’s solution and incubated at 60 °C for 1 h. Slides were allowed to cool to room temperature for 10 min, rinsed well in running tap water, and rinsed in deionized water. Slides were immersed in freshly prepared Weigert’s iron hematoxylin for 10 min, washed in running tap water, and rinsed in deionized water. Slides were immersed in the Gomori one-step aniline blue trichrome solution for 20 min followed by differentiation in 0.5% acetic acid for 2 min and quickly rinsed in deionized water before dehydration, clearing, and mounting, as listed above.

#### 2.3.4. Oil Red O (ORO) Staining

Formalin-fixed slides were rinsed in deionized water. Excess water was blotted from each slide prior to being immersed in 100% propylene glycol on a shaker with gentle, continuous agitation for 5 min. Slides were transferred directly to Oil Red O propylene glycol solution (Newcomer Supply, Middleton, WI, USA) and stained with gentle, continuous agitation for 1 h. Slides were differentiated in 85% propylene glycol with gentle, continuous agitation for 3 min followed by gentle rinsing in two changes of deionized water. Slides were counterstained in hematoxylin for 2 min, gently washed in tap water, blued in Scott’s tap water for 1 min, gently rinsed in two changes of deionized water, and mounted with Gel/Mount aqueous mounting media (Biomeda, San Jose, CA, USA) and a glass coverslip. Edges of the glass coverslip were sealed with clear fingernail polish to prevent evaporation of the aqueous mounting media.

### 2.4. Quantification of Liver Lipid Content

Lipid was isolated from the dried liver samples (*n* = 10 per group) using a methyl tert-butyl ether (MTBE) biphasic extraction method [28]. Weights collected throughout sample processing (fresh tissue, desiccation, extraction, lipid isolation, and dry matter) were used to quantify lipid content expressed as mg of lipid per mg of dry liver weight.

### 2.5. RNA Isolation and RNA-Seq Analysis

Frozen liver samples (*n* = 10 per group) were ground to a fine powder using ceramic pestles in ceramic mortars filled with liquid nitrogen. RNA samples were extracted using the RNeasy mini-kit (QIAGEN, Inc., Germantown, MD, USA) according to the manufacturer protocol. RNA integrity was determined with Experion automated electrophoresis station (Bio-Rad Laboratories, Inc., Hercules, CA, USA). Samples were diluted to a concentration of 4 ng/µL in the 50 µL of RNase-free water and under dry ice shipped to the Genomics and Microarray Core at the University of Colorado Anschutz Medical Center (Aurora, CO, USA) for the cDNA library construction and preparation using Nugen Universal mRNA kit (Tecan Genomics, Inc., Redwood City, CA, USA) and subsequent paired-end (2 × 150 bp) RNA sequencing (RNA-Seq hereafter) using a NovaSEQ 6000 sequencer (Illumina, Inc., San Diego, CA, USA). The sequencing data were further processed using QIAGEN CLC Genomics Workbench, version 21.0.3 (QIAGEN, Redwood City, CA, USA), https://digitalinsights.qiagen.com (accessed on 24 April 2021). Raw reads were trimmed using quality scores (quality limit = 0.05) for removal of ambiguous nucleotides and read-through adapters. The resulting high-quality reads were mapped to *Mus musculus* genome reference, version GRCm38, using gene ontology associations to generate transcript level expression tracks. The transcript expression data containing 66,624 genes were subjected to the differential abundance analysis (see Section 2.6) producing output datasets of pairwise comparison pairs (35% vs. 0%, 70% vs. 0%, and 70% vs. 35% bean diet groups). Differentially expressed genes (DEGs), their *p*-values, *p*-values corrected for multiple comparisons using Bonferroni method and Benjamini–Hochberg procedure controlling the false discovery rate (FDR; *q*-values), expression fold change, expression log_2_ ratios, and the values for the maximum of the two average reads per kilobase million (RPKMs) per diet group in a comparison pair comprised each comparison table. DEGs containing at least 10 RPKMs for each comparison pair were uploaded to QIAGEN Ingenuity Pathway Analysis (IPA) software, v81348237 (QIAGEN, Redwood City, CA, USA), https://digitalinsights.qiagen.com/IPA (accessed on 13 July 2022) [29], for further analysis.

Within IPA, each comparison dataset was subjected to the Core Analysis with Ingenuity Knowledge Base (Genes only) as a reference set and relaxed integral filters for species, tissues, and cell lines. Expression log_2_ ratio was selected as a measurement to calculate directionality in the analysis. To maximize the biological interpretation, the list of DEGs was further quality-filtered by the *p*-value < 0.05 to keep each comparison pair dataset within recommended by IPA limits (Table 1). IPA mapped DEGs to identifiers (IDs); 9 IDs in each group were unmapped and discarded. Identifiers that mapped to the same molecule were resolved to one data measurement by the expression *p*-value.

Canonical Pathways, Upstream Analysis, and Diseases and Functions engines were used to analyze DEGs. Briefly, expression patterns of the DEGs were cross-referenced with the published studies worldwide from the QIAGEN Ingenuity Knowledge Base, and *p*-values of overlap between the observed DEGs patterns and their associations were calculated. Activation *z*-scores measuring the prediction strength of the DEGs effects (activation or inhibition) were calculated based on direction of DEGs expression changes. Mapped functional associates were deemed significant with the |*z*|-score ≥ 2 and the *p*-value < 0.05 of the overlap (B-H *p*-value < 0.1) between our experimentally observed dataset and the QIAGEN Ingenuity Knowledge Base.

### 2.6. Statistical Evaluation

Hepatic lipid quantification data (expressed as milligrams per dry liver tissue weight) were analyzed and visualized in *R* (v4.2.2) using *FSA*, *rstatix*, and *gpubr* packages.

Statistical differential expression analysis of RNA-Seq data in CLC Genomics Workbench was conducted through the Differential Expression for RNA-Seq tool via generalized linear modeling for each gene assuming the negative binomial distribution of read counts and subsequent Wald testing for all group pairs to generate the expression track tables. In IPA, *p*-values of overlap were calculated using a right-tailed Fisher’s exact test and further adjustment for multiple hypothesis testing applying Benjamini–Hochberg correction method (B-H *p*-values). At times, *p*-values were reported in their negative logarithmic format (−log*_p_*_-value_; −log_B-H *p*-value_) as provided by IPA. *z*-scores were calculated using internal IPA algorithms.

Selected DEGs were visualized in *R* in counts per million (CPMs) normalized for the library sizes with the trimmed mean of M values (TMM) method using *ggpubr* package.

## 3. Results

### 3.1. Livers Accumulate Less Lipid upon Increasing Consumption of Bean

**Figure 1:**
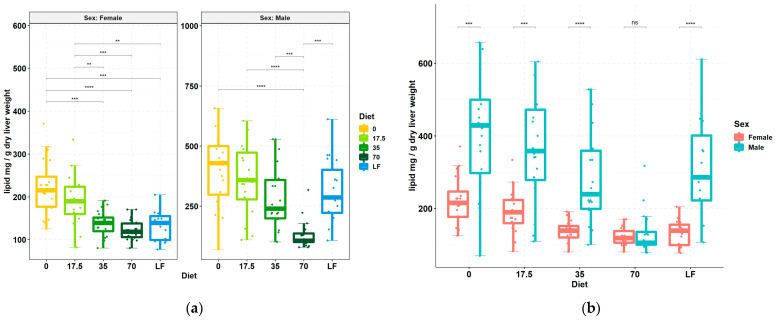
Effect of dietary bean on the liver lipid content. Box plots report amount of hepatic lipid (in mg) normalized to g of dry liver weight across the diet groups and sex cohorts. Diets are indicated by the percent (%) of dietary protein provided by bean, including a low-fat (LF) diet as a negative control. Kruskal–Wallis testing showed significant differences by the diet effect (χ^2^ = 41.598, *p*-value = 2.021× 10^−8^ in the female cohort; χ^2^ = 40.15, *p*-value = 4.029 × 10^−8^ in the male cohort). (**a**) Pairwise comparisons between the diet group were conducted using the post-hoc Dunn test; (**b**) Pairwise comparison between sex cohorts were tested with the Mann–Whitney *U* test. The Benjamini–Hochberg method was used for the multiple testing correction of *p*-values. ** *p*-value < 0.01; *** *p*-value < 0.001; **** *p*-value < 0.0001.

MAFLD onset is grossly identified by excessive accumulation of lipid in the liver. Consumption of bean markedly reduced the lipid content per g liver dry weight in both females and males (Figure 1). Regression analysis indicated that the reduction was linear across increasing bean doses in both sexes (regression coefficient, −35.3, *p* < 0.001 and −90.3, *p* < 0.001 in female and male mice, respectively). Pairwise comparisons revealed that females exhibit less hepatic lipid at the 35% dose without any statistical distinction with the higher bean dose, whereas males had a significant effect only in the 70% group which statistically differed from both 0% and 35% bean doses (Figure 1a). The 70% bean dose nullified sex-dependent differences in hepatic lipid levels (Figure 1b).

### 3.2. Dietary Bean Visually Reduces Steatosis Severity in the Liver Tissue

**Figure 2:**
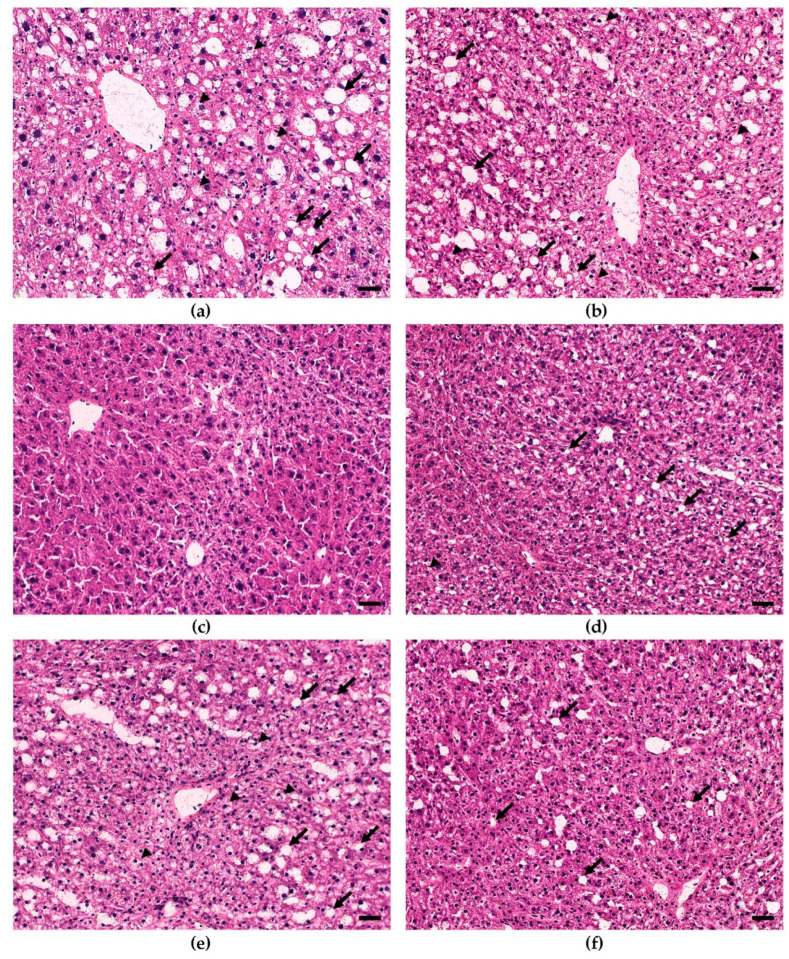
Comparison of lipid accumulation in male and female H&E-stained liver sections across different diets. (**a**) Male 0%; (**b**) Female 0% bean; (**c**) Male 70% bean; (**d**) Female 70% bean; (**e**) Male low fat; (**f**) Female low fat. Steatosis (arrows); hepatocyte ballooning (arrowheads); magnification 200×; bars = 40 µm.

**Figure 3:**
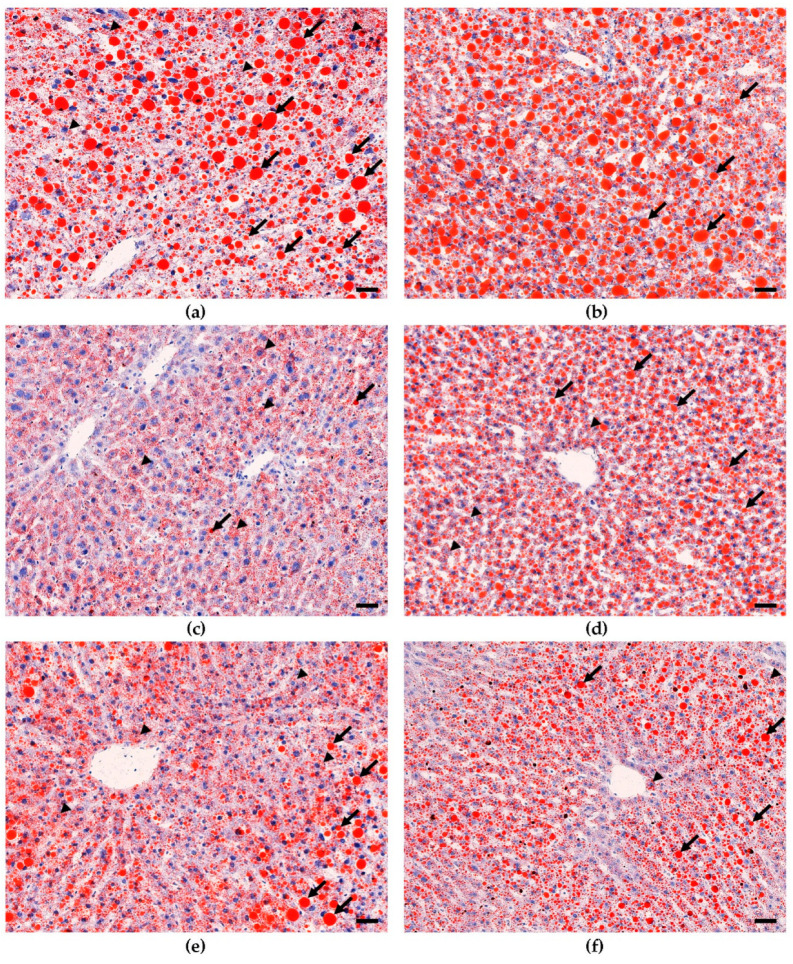
Comparison of lipid accumulation in male and female Oil Red O-stained liver sections across different diets. (**a**) Male 0%; (**b**) Female 0% bean; (**c**) Male 70% bean; (**d**) Female 70% bean; (**e**) Male low fat; (**f**) Female low fat. Extracellular lipid (arrows); intracellular lipid (arrowheads); magnification 200×; bars = 40 µm.

To determine the phenotypic liver response to consumption of increasing doses of bean, frozen sections of liver were stained for histological evaluation. The H&E-stained sections (Figure 2) showed that livers from male and female 0% bean-fed mice displayed the classic features of hepatic steatosis, i.e., extensive accumulation of lipid droplets, heterogenous in size and shape, in the cytoplasm and the consequential ballooning [8]. However, only focal areas of lymphocyte accumulation were detected, and no evidence of fibrosis was observed. These changes were not observed in mice fed the 70% bean diet, and lipid accumulation was similar to that observed in the low-fat-fed group of mice. To confirm these effects, sections were subjected to ORO staining (Figure 3) and to trichome staining. The ORO staining confirms the results of the H&E stain relative to lipid accumulation. The trichrome stain (data not shown) confirmed the lack of collagen accumulation in any of the animals evaluated.

### 3.3. Liver Transcriptomic Signature upon Consumption of Bean Indicates Protection from Hepatotoxicity

**Figure 4:**
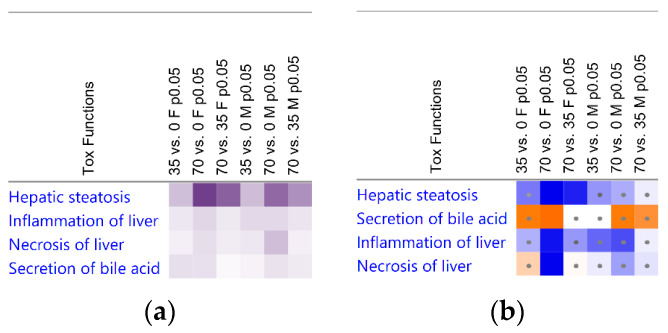
Heatmap of the Hepatotoxicity Functions based on the differentially expressed gene patterns in the liver tissue samples of female (F) and male (M) mice. Functions were organized by the collective score values. Pairwise comparisons are indicated in the column headers with first three from the left for the females and the other three columns for the males. Significant functions were filtered by B-H *p*-values < 0.1 and |*z*|-scores > 2, (**a**) shades of purple indicate −log_B-H *p*-values_; (**b**) blue shades indicates inhibition, orange—activation, for 70% diet group in at least one sex cohort. Dots denote insignificant |*z*|-scores < 2.

To infer the underlying molecular mechanisms that may explain the effects on liver lipid content and histology (Figure 1, Figure 2 and Figure 3), total RNA was isolated from liver samples and subjected to the RNA-Seq. Only 0%, 35%, and 70% bean-containing diet groups were selected as the most prominently responsive to dietary beans. The Tox Functions engine within the IPA Core Analysis was used to provide an overview of the toxicity endpoints associated with the bean-induced liver within the Hepatotoxicity category as a focus. Accordingly, bean-induced DEGs patterns revealed significant suppression of hepatic steatosis, inflammation, and necrosis, and upregulation of bile acid secretion in both female and male mice (Figure 4a). Suppression of hepatic steatosis was significant in the 70% compared to both the 0% (*z*-score = −2.549) and the 35% (*z*-score = −2.195) bean groups in females (Figure 4b). In contrast, the bean-stimulated expression patterns in the male cohort did not reach statistically significant *z*-score values but the trend was similar to the females (Supplementary Table S3).

Collectively, hepatotoxicity analysis of global bean-induced liver transcriptomes indicates that consumption of bean altered expression of hepatic genes predominantly related to the lipid metabolism, and it was more pronounced in female than male mice—an observation consistent with hepatic lipid content in the bean-fed animals (Figure 1) and the histopathologic changes shown in Figure 2 and Figure 3. Therefore, we next focused on the key mechanisms regulating hepatic FA metabolism involving the observed bean-induced DEGs.

### 3.4. Bean Consumption Maps to Hepatic Fatty Acid Metabolic Pathways and Regulators

**Figure 5:**
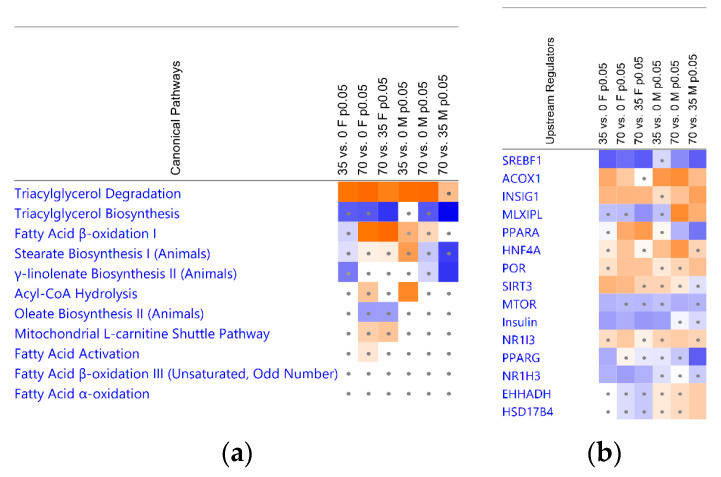
Canonical pathways and upstream regulators associated with fatty acid metabolism in liver upon consumption of common bean. (**a**) Canonical pathways; (**b**) upstream regulators. Pairwise diet comparison groups are indicated in the columns. Heatmap colors and their intensity indicate activity status according to the *z*-score values direction: orange signifies activation and blue—inhibition. Dots denote insignificant |*z*|-scores < 2. Indicated pathways had a significant *p*-values of overlap < 0.05 (B-H *p*-values < 0.1) and are organized by the collectively largest absolute *z*-score values across the comparisons.

Hepatic fat is stored as lipid droplets which consist predominantly of triacylglycerols (TAGs) formed by esterification of free fatty acids (FAs) with glycerol [30]. Considering the central role of FAs to MAFLD progression [31,32], we performed IPA Canonical Pathways analysis with a particular focus on metabolic signaling associated with FAs. Consumption of beans significantly upregulated TAG degradation and downregulated TAG biosynthesis (Figure 5a). The FA β-oxidation was upregulated in the 70% dose compared to the 0% and 35% bean groups in females, albeit the FA activation and the mitochondrial L-carnitine shuttle pathway had significant overlaps with the observed DEGs in both cohorts but insufficient *z*-scores to infer their activation (Figure 5a; Supplementary Table S3). Similarly, β-oxidation of unsaturated/odd-number FAs and the FA α-oxidation also displayed only significant overlap with bean-induced DEGs (Figure 5a; Supplementary Table S3). Finally, biosynthetic pathways of long-chain FAs conjugates oleate, stearate, and γ-linoleate indicated a pattern of inhibition, which corresponds to suppression of lipogenesis upon consumption of beans for both sex cohorts (Figure 5a).

To identify the most significant potential regulators of bean effects in the context of lipid metabolism, we subjected the observed DEGs to the Upstream Analysis in IPA. The sterol regulatory element-binding transcription factor 1 (SREBF1; also known as sterol regulatory element-binding protein 1c, SREBP1c) and MLX-interacting protein-like (MLXIPL; also known as the carbohydrate regulatory element-binding protein, ChREBP) are the key transcriptional regulators of *de novo* lipogenesis in liver. SREBP1 was predicted to be inhibited in all but the lowest bean dose groups. MLXIPL showed a trend of inhibition in the female cohort; however, in males, was upregulated in the 70% versus 0% and 35% bean diets (Figure 5b). While SREBF1 is stimulated by insulin (even under conditions of insulin resistance) via mammalian target of rapamycin (mTOR) and by liver X receptor α (LXRα, encoded by *Nr1h3*), MLXIPL signaling is induced by dietary glucose (even more potently by dietary fructose) independently of insulin or dietary fat [33,34,35]. Accordingly, we observed inhibition of insulin and its receptor INSR and insulin receptor substrate 1 (IRS-1) based on bean-induced downstream DEGs indicative of improved insulin sensitivity. To support the trend, mTOR complex 2 partner and regulator RICTOR (rapamycin-insensitive companion of mTOR) displayed inhibition, whereas negative regulators of mTOR complex 1, tuberous sclerosis proteins TSC1/2, were upregulated (Supplementary Table S4). SREBF cleavage-activating protein (SCAP) promoting SREBF1 activity is counteracted by insulin-induced genes INSIG-1/2—also in accordance with our data (Figure 5b). LXRα belongs to the nuclear receptors [36], and bean-induced DEGs predict its inhibition, consistent with upregulation of nuclear receptor co-repressor N-Cor (Figure 5b) which also suppresses their target genes [37]. Another nuclear receptor group, peroxisome proliferator activated receptors (PPARs) are involved in regulation of majority of genes involved in lipid metabolism as nutrient sensors activated by FA derivatives [34,36]. Hepatic PPARα (*Ppara*) stimulates expression of genes associated with mitochondrial and peroxisomal FA β-oxidation, whereas PPARγ (*Pparg*) is associated adipose tissue and lipid droplet regulation but shows strong responsiveness to MAFLD in liver. Bean suppressed PPARγ, but PPARα was predicted to be upregulated in females yet downregulated in males based on their downstream DEGs. Progression of MAFLD to MASH involved regulatory network of upregulated SREBF1, PPARγ, and disrupted activity of hepatocyte nuclear factors HFN1A, HFN4A, and ONECUT1 [38,39] which were predicted to be upregulated upon bean consumption (Figure 5b, Supplementary Table S4). Finally, sirtuin 3 (SIRT3) improves metabolic disfunction, and its activation has been associated with beneficial caloric restriction and exercise [33]; however, here, isocaloric diet containing even 35% protein deriving from bean was sufficient to arrange DEGs patterns predicting activation of SIRT3 in females (Figure 5b).

Taken together, dietary bean dose-dependently affects key regulatory nodes of TAG metabolism associated with MAFLD, and such effects differ between females and males.

### 3.5. Consumption of Beans Reduces Uptake and De Novo Lipogenesis of Free Fatty Acids and Differentially Affects Transport, Oxidation, and Incorporation into Lipid Droplets

**Figure 6:**
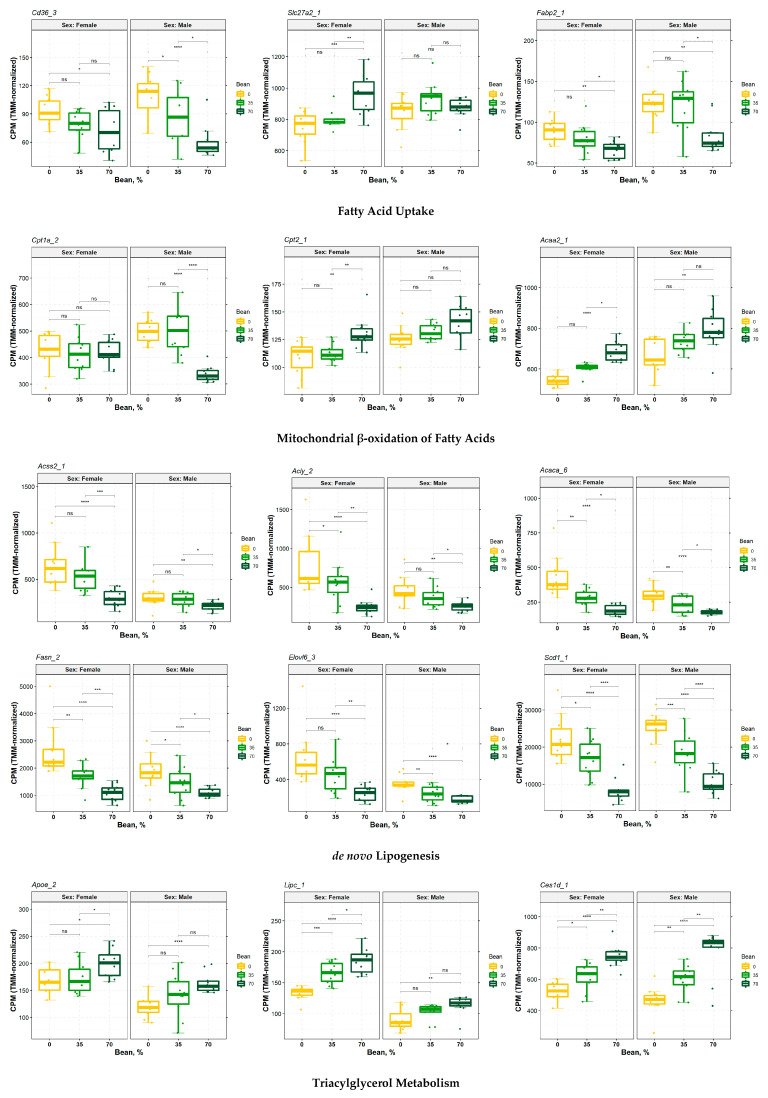
Expression of genes involved in lipid metabolism in the livers of female and male mice upon consumption of beans. Differential expression analysis performed in CLC Genomic Workbench. * *p*-value < 0.05; ** *p*-value < 0.01; *** *p*-value < 0.001; **** *p*-value < 0.0001.

TAGs serve as both the source and preferred anabolic end-point of intracellular FAs [30,34]. Corresponding to Canonical Pathways analysis results in Figure 5a, dietary bean increased expression of TAG hydrolases, such as hepatic lipase C (*Lipc*) and several carboxylesterases *Ces1d*, *Ces2a*, *Ces2e*, *Ces3a*, *Notum*, etc., which signify greater capacity for lipolysis in the liver in female and male livers. Upstream analysis indicated that males’ DEGs predicted activation of hormone-sensitive lipase (*Lipe*) and monoacylglycerol lipase (*Mgll*), despite observed reduced expression of *Mgll* and *Pnpla2*—lipolysis-initiating lipase which was slightly expressed more in females fed beans, instead (Figure 5b; Supplementary Figure S1). TAGs can also undergo lipophagy via formation of autolysosomes [34,40]. While both cohorts had reduced expression of lysosomal acid lipase A (*Lipa*), females also increased expression of microtubule-associated protein 1 light chain 3 (LC3; encoded by *Map1lc3a;* Supplementary Figure S1), one of the markers of lipophagy (autophagic degradation of lipid droplets) in liver [40,41,42,43]. Nevertheless, bean-induced DEGs showed inhibition of upstream transcription factor EB (TFEB; Figure 5b), which regulates lipophagy and drives expression of LIPA, serving as another piece of evidence that lipophagy is hardly mediating dietary bean effects [42,44]. Finally, bean-fed females increased expression of LDLR-related protein 1 (*Lrp1*) and apolipoprotein E (ApoE), which are involved in uptake of dietary TAG-rich chylomicron remnants and improved MAFLD (Figure 6; Supplementary Figure S1 and Table S4) [45].

Lipolysis leads to intracellular FA flux, which in MAFLD patients is predominantly driven by uptake of free non-esterified FAs [46]. Consuming increasing doses of beans reduced expression of the plasma membrane FA-binding protein (FABPpm, encoded by *Fabp2*) and FA translocase/cluster of differentiation 36 (CD36)—higher levels of which are a biomarker of high-fat diet-induced hepatic steatosis [34] and whose reduction had a larger magnitude in males than in females (Figure 6). Expression of *Cd36* is regulated by PPARγ, aryl hydrocarbon receptor (AHR), LXRα, mTOR [34,47], and even SREBF1 and MLXIPL [34,48], all of which were affected by dietary bean based on their downstream DEGs (Figure 5b; Supplementary Table S6). Females also slightly increased expression of the very long-chain FA transport proteins 2 and 5 (FATP2/5, also known as very long-chain acyl-CoA synthetases and solute carrier family 27A members 2/5, encoded by *Slc27a2/5*), despite significant downregulation of SLC27A2 in upstream analysis (Figure 5b and Figure 6). However, *Slc27a5*, which was the only upregulated FATP in males, has been reported to be rather associated with conjugation of bile acids than FAs uptake (Supplementary Figure S2 and Table S4) [49].

Once inside the cells, FAs are transported by cytosolic FA-binding proteins (FABPs), acyl-CoA-binding domain containing proteins (ACBDs), and others [34]. Males exhibited reduced expression of *Fabp4/5* and *Acbd4*, whereas females decreased expression of *Fabp5* and *Acbd5* with a slight increase in *Fabp1* levels (Supplementary Figure S2 and Table S4). While all these FABPs correlate with the hepatic fat content [35], FABP1 was also associated with protective role by redirecting intracellular FAs towards oxidation, energy utilization, and disposal [33,50,51].

FAs enter metabolic pathways following activation by acyl-CoA synthetases (ACSs) as respective acyl-CoA molecules [52]. Consumption of beans suppressed short-chain ACS *Acss2* and long-chain ACS *Acsl5* in both sex cohorts (females also reduced levels of ER-based *Acsl3*). In contrast, bean increased propionyl-CoA ACS *Acsf*, medium-chain ACSs *Acsm1/5* (however, *Acsm3* was reduced in females), and expression of long-chain *Acsl1*. While *Acsl1* is associated with oxidation of long-chain FAs, *Acsl3/5* are mainly connected to thioesterification of *de novo* synthesized FAs for lipogenic pathways (Supplementary Figure S3 and Table S4) [34,51].

Oxidation is the main mechanism of FAs utilization which occurs predominantly in mitochondria (β-oxidation), but also can take place in peroxisomes (α-oxidation of branched-chain FAs and β-oxidation of very long-chain FAs) and in the endoplasmic reticulum (ER; ω-oxidation of FAs via cytochrome P450 enzymes, CYPs) [52]. The 70% of bean sample compared to the bean-free control and lower bean dose in males significantly reduced expression of carnitine palmitoyltransferase CPT1 (*Cpt1a*)—the rate-limiting step of mitochondrial β-oxidation—regulating long-chain FAs transfer across mitochondrial outer membrane. In contrast, females elevated carnitine-acylcarnitine translocase (CACT, *Slc25a20*) and CPT2 levels mediating transport of CPT1-derived acyl-carnitines across inner mitochondrial membrane and conversion thereof back to acyl-CoAs, respectively [48,52]. CPT2 also showed a trend of increase in the male cohort but without significant *p*-value (Figure 6; Supplementary Table S4 and Figure S4). CPT1 is regulated by MLXIPL, PPARα, and PPARγ coactivator-1 (PGC-1), and subunit and stabilizator of the latter, *Ppargc1b*, showed inhibited trend in upstream analysis (Supplementary Table S6) [34,50,53]. Subsequently, bean consumption differentially affected enzymes involved in the course of FA β-oxidation in mitochondria with a noticeable trend towards an increased acyl-CoA dehydrogenases (ACADs) for short-chain FAs *Acadsb*, medium-chain FAs *Acadm*, and long-chain FAs *Acadl*, *Acadvl*, and *Acad11* in females, whereas males upregulated only short-chain FAs mitochondrial ACADs *Acadsb* and *Acad8*. Females also upregulated 3-hydroxyacyl-CoA dehydrogenase *Hadh*, including hydroxyacyl-CoA dehydrogenase-α (*Hadha*) and hydroxyacyl-CoA dehydrogenase-β (*Hadhb*)—units of the mitochondrial trifunctional protein degrading long-chain FAs [54]; as well as final thiolase (*Acaa2*) producing acetyl-CoA and shorter by two carbons acyl-CoA molecules [52]. Additionally, enoyl-CoA isomerase (*Eci2*) and 2,4-dienoyl-CoA reductase (*Decr1*), both of which handle unsaturated FAs, were induced in females (Supplementary Figure S4 and Table S4). In contrast, males upregulated only mitochondrial short-chain FAs ACADs (*Acadsb* and *Acad8*), *Acaa2*, and *Eci2*. Such a transcriptomic signature implies that despite a greater capacity of female livers to β-oxidize various FAs, males rather target short-chain FAs in mitochondria (Supplementary Figure S2).

Long- and very long-chain acyl-CoAs canonically undergo initial catabolism via peroxisomal β-oxidation which consists of essentially the same reactions as the mitochondrial one but catalyzed by different enzymes [52,55,56]. Consumption of beans induced expression of peroxisomal acyl-CoA oxidase (*Acox1* for females and *Acox2* for males) mediating rate-limiting initial dehydrogenation. Upstream analysis indicated that ACOX1 was predicted to be upregulated in both sex cohorts upon bean consumption with higher *z*-score in males than females (Supplementary Figure S5 and Tables S4 and S6). L-bifunctional protein (LBP, encoded by *Ehhadh*) and D-bifunctional protein (DBP, encoded by *Hsd17b4*) mediating hydration and subsequent dehydrogenation of FAs were upregulated (Figure 5b) in males, but peroxisomal 2,4-dienoyl-CoA reductase *Decr2* expression was reduced. Subunits of final thiolases in peroxisomes *Acaa1a/b* were increased only in female cohort. Finally, there was a slight decrease in peroxisomal FAs transporter—ATP-binding cassette (ABC) protein of subclass D *Abcd1* in both sex cohorts under bean-containing diets. This is in contrast to increased *Slc27a2* and *Acbd5* associated with peroxisomal import of long- and very-long chain FAs in females (Supplementary Figure S5) [55]. 

Imbalanced hepatocytes upon MAFLD counterintuitively respond to systemic lipid overload with enhanced *de novo* lipogenesis of new FAs [33,34,35]. Bean consumption incrementally reduced expression of key enzymes in this process: aforementioned *Acss2* and ATP-citrate lyase (ACLY), both cytosolic and mitochondrial acetyl-CoA carboxylases I and II, respectively (ACC1/2, *Acaca/b*), fatty acid synthase (FASN), including elongases *Elovl2/5/6* and desaturases *Fads1/2*, *Scd1* in both sex cohorts. Males additionally decreased levels of *Elovl1* transcripts but increased such of *Elovl3*, whereas females reduced expression of lipogenic *Slc25a1* (Figure 6; Supplementary Figure S6 and Table S4). Expression of these lipogenic genes is regulated mainly by SREBF1, especially when stimulated by postprandial circulating insulin, and by MLXIPL induced by dietary carbohydrates [8,35].

Newly synthesized long-chain FAs are in the form of acyl-CoAs which can enter TAGs biosynthetic pathways or be first transformed to free FAs acyl-CoA thioesterases (ACOTs). The glycerol-3-phosphate (G3P) pathway (also termed the Kennedy pathway; involves G3P- (GPATs) and acyl-G3P acyltransferases (AGPATs)), while 2-monoacylglycerol pathway consists of mono- (MGATs) and diacylglycerol acyltransferases (DGATs). Both are connected by lipins-mediated production of diacylglycerol [34,48]. Bean consumption reduces expression of lipins *Lpin2*, a GPAT encoded by *Gpam*, AGPATs *Agpat2* (females) and *Agpat3* (males) [34]. Males also reduce glycerol kinase *Gk* that generates G3P from glucose and glycerol but upregulate *Dgat2*. Additionally, bean induced significant downregulation of *Lpcat3* (both sex cohorts) and *Pnpla3* (females only) that possess acyl-G3P acyltransferase activity within TAGs biosynthesis pathway according to IPA Canonical Pathways (Figure 5a; Supplementary Figure S7). Finally, packaging of newly made TAGs into lipid droplets also involves DFF45-like effector proteins (CIDE group) together with abovementioned ACSL3/5 [34], and consumption of beans reduced their expression in males (Supplementary Figure S7), while females’ DEGs indicated downregulation of CIDEC in the upstream analysis (Figure 5b). CIDE proteins, ACSL3, DGAT2, including perilipins (PLINs), PNPLA3, and apolipoproteins are packaged into lipid droplets for storage and very low-density lipoproteins (VLDLs) for export [57]. Consumption of bean reduced expression of *Plin3* (males also reduced *Plin2*), apolipoproteins ApoA4 and ApoC2 (females also reduced ApoA5) but increased levels of *Plin5* as well as aforementioned ApoE (females also elevated ApoA2, while males increased ApoH; (Supplementary Figure S7). The upstream analysis also indicated that bean-upregulated FOXA1 (Figure 5b; Supplementary Table S6) which suppresses multiple target genes involved in TAGs synthesis, such as GPAM, DGAT2, and some apolipoproteins [58].

Ultimately, increasing dose of consumed beans significantly affected genes involved in every key mechanism of hepatic FAs metabolism, enabling its effective regulation of hepatocellular FAs and TAGs levels. These findings were consistent with results of IPA Canonical Pathways analysis, whereas IPA Upstream analysis revealed similar patterns of bean-induced DEGs and their functional association.

## 4. Discussion

MAFLD is a global public health concern rooted at the crossroads of metabolically dysregulated chronic non-communicable diseases and the respective detrimental economic, societal, and environmental consequences [59,60,61]. Effective care of MALFD patients is limited by the affordability, accessibility, and safety of interventions for the public and depends on the stage of disease progression. Nevertheless, even if better treatment measures are developed, modifying lifestyle risk factors remains central in MAFLD management, therapy, and prevention, according to consensus statements and recommendations for the public health agenda from the multidisciplinary group of experts [5]. Within lifestyle, chronic positive energy balance, particularly in adults, is a strong driver of MAFLD, but the global trends have been opposite and highly refractory to change [62]. This reality drives attention toward global food patterns and food types therein.

Given that the Mediterranean-type food pattern is reported to be protective against metabolic disorders, such as MAFLD, whereas the Western-type pattern is a major risk factor for the disease, a category of foods discriminating the two patterns are pulses. Moreover, dietary guidelines, such as The Dietary Guidelines for Americans [14], inadvertently support the consumption of pulses as a vegetable side dish rather than as a staple food. We argue that as a consequence of such exclusion, dietary quality and associated health benefits are markedly reduced. This dilemma is partly due to a lack of clinical or population data that inform what dose/dietary concentration of pulse is needed to secure protection against fatty liver disease and whether biological sex plays a role. These issues are directly addressed by the work reported herein.

### 4.1. Dietary Bean-Induced Liver Phenotype

The design of this study used *ad libitum* feeding of a recognized obesogenic diet formulation that induces both MAFLD as well as obesity in C57BL6/J mice since that is the human context in which our study questions are most appropriately considered. Figure 1, Figure 2 and Figure 3 clearly show that excessive hepatic steatosis—a hallmark of MAFLD [8,27,63]—is prevented by common bean consumption regardless of sex. Such an effect only becomes uniform enough to achieve statistical significance in female mice when ≥ 35% of dietary protein is derived from bean. In male mice, 70% dose is required to render an effect statistically distinct from the bean-free control. Implication of hepatic steatosis in the response to the dietary bean was consistent with the results of the bean-induced DEGs that mapped to this hepatotoxicity with the highest *p*-values (Figure 4). The magnitude of bean required relative to the amount currently consumed in populations such as those in developed countries such as the USA, Canada, and many countries in Europe, underscores the critical importance of understanding the metabolic pathways through which common bean exerts these effects.

### 4.2. Triacylglycerol as the Main Chemical Form of Hepatic Lipid

Hepatic steatosis develops as acquisition and biosynthesis of intrahepatic lipids surpass the limits of their catabolism, utilization, and export such that excess fat is stored in intracellular vacuoles as lipid droplets [35]. The latter consist of neutral lipids, e.g., TAGs and cholesterol esters, surrounded by a phospholipid monolayer carrying proteins involved in synthesis, degradation, and trafficking of those lipids [57]. There is a fine-tuned balance between formation and degradation of TAGs within lipid droplets. TAGs are the preferred form of energy storage in the liver as they are biochemically inert [30] and, thus, serve as both the source and anabolic end-point of hepatocellular FAs [34]. In contrast, diacylglycerols, acyl-carnitines, ceramides, glycerophospholipids, sphingolipids, free cholesterol, and especially free FAs are associated with severe lipotoxicity [31,32,64]. About 15% of MAFLD-associated TAGs in the liver originate from the diet [46]. Females upregulate *Lrp1* expression, indicative of greater uptake of chylomicron remnants but, simultaneously, of improved MAFLD state [45]. Transcriptomic analysis revealed that the consumption of beans significantly upregulates canonical degradation of TAGs with the concomitant pattern of TAGs biosynthesis inhibition (Figure 5a). Therefore, the expected reduction in hepatic TAGs content based on such a transcriptomic signature is also observed in the lipid quantification and liver histopathology (Figure 1, Figure 2 and Figure 3).

### 4.3. Contribution of Extracellular Free Fatty Acids Uptake

Excessive caloric intake renders adipose tissue and skeletal muscle (the primary destinations of dietary fat in the postprandial state) overloaded with intestinally derived chylomicrons so that their remnants and their spillover-derived non-esterified FAs are cleared from circulation by the liver [34]. Moreover, the accompanying dysfunction of adipose tissue increases its rates of lipolysis [32,65], releasing non-esterified free FAs into circulation. These free FAs are the primary source for 59% of TAGs in MAFLD livers. MAFLD-compromised hepatocytes upregulate the uptake of free FAs [34,50,64]. While short- and medium-chain FAs diffuse across hepatocyte plasma membrane due to their lipophilicity [66], long-chain FAs entry is mediated mostly by the membrane-bound proteins [34]. Higher levels of respective CD36 are a biomarker of high-fat diet-induced hepatic steatosis [34], and its reported knockdown improved insulin sensitivity and steatosis owing to observed decrease in FA uptake and TAGs concentration [34,50,67]. CD36, which was dramatically reduced more in males than females upon dietary bean, is considered the most contributing factor in FA-induced lipotoxicity in MALFD [64]. Its expression is regulated by PPARγ and LXR [34], whose downregulation, according to upstream analysis, was consistent with CD36 suppression (Figure 5b). Therefore, upon bean consumption, females show a greater response in mediating uptake of lipoproteins and their degradation, whereas males rather downregulate FAs transport mechanisms.

### 4.4. Hepatocellular Oxidation of Fatty Acids

Analysis of intracellular FAs transport proteins and FABPs, ACSs, and ACOTs, as well as transcripts of FA oxidation enzymes indicated: a) bean consumption generally exerted a pro-catabolic profile in both sex cohorts; b) females displayed more evidence for enhanced mitochondrial β-oxidation of long-chain FAs and peroxisomal β-oxidation of very long-chain FAs; and c) males’ DEGs patterns connect more to mitochondrial β-oxidation of short-chain, odd-number, and unsaturated FAs and peroxisomal β-oxidation of long-chain FAs. AMP-activated protein kinase (AMPK) suppressing mTOR, SREBP1, and ACC1/2 in favor of FAs β-oxidation [50,68] showed patterns of bean-induced activation (Supplementary Table S6), which is in accordance with the sex-dependent signature of bean consumption that significantly mapped FAs β-oxidation to females. Taken together, this points to a priori differential dietary challenges and sex-dependent differences in hepatic lipid catabolism which accordingly result in differential effects of dietary beans. While females exhibit a more pronounced increase in capacity for FA oxidation in livers than males, the latter demonstrate differential effects of bean consumption on hepatic FA β-oxidation.

### 4.5. Bean Effects Shared by Both Sex Cohorts

A dramatic reduction in the capacity to drive *de novo* lipogenesis in hepatocytes of mice was more uniform between females and males. Considering that mice fed bean-containing diets also reduced body adiposity [22], the contribution of adipose tissue-derived free FAs would be markedly reduced. Furthermore, under the MAFLD conditions, 26% of hepatic TAGs originate from *de novo* lipogenesis of new FAs in hepatocytes [46]. Thus, the suppression of lipogenic genes we demonstrate herein, including upstream regulators assessment indicating significant and dose-dependent inhibition of lipogenic SREBF1, potentially resembles the primary mechanism of bean-induced protection. After all, bean-promoted health benefits were potent enough to reduce expression of genes driving the obesogenic phenotype in C57BL6/J mouse model, such as *Acss2*, *Acly*, *Elovl6*, *Scd1*, *Acaca*, and *Slc25a1* [26]. Therefore, consumption of beans induces sex-dependent effects on improving hepatic FA oxidative capacity but not on the suppression of *de novo* lipogenesis. This is consistent with observed transcriptomic trend of TAGs biosynthesis inhibition (Figure 5a).

### 4.6. Differential Sex-Dependent Effects of Common Bean

The multi-omics integrative studies determined that males exhibit more MAFLD-associated hepatic genes than females, especially those associated with lipid metabolism, and identified key genes driving MAFLD in the livers of female and male mice, including but not limited to *Insig1*, *Acss2*, *Acot/4*, *Pnpla3* shared by both sexes; female-specific *Lipc*, *Srebf1*, *Slc27a5*; male-specific *Cidec*, *Mogat1*, *Cd36*, *Fasn*, *Cpt2* [69,70]. Consistently, all these genes were detected among observed DEGs or their predicted upstream regulators, including the observation that males displayed more hepatic DEGs upon bean consumption than females. Observation that bean-induced protective effects from hepatic steatosis were qualitatively observed at the lower dose in females compared to males indicate that there are sex-dependent differences in MAFLD etiology and that males overall exhibit greater susceptibility to MAFLD comorbidities [69,70,71,72]. We report that female and male mice responded to dietary beans with opposite effects on PPARα and MLXIPL, and males exhibited steatogenic associations (Figure 5b; Supplementary Table S4 and S6). Levels of PPARα and PPARγ are increased upon a high-fat diet; however, knockout studies indicated that PPARα ablation exacerbates MAFLD and PPARγ ablation protects mice from its development [36]. MLXIPL contribution to MAFLD development stems from obesogenic effects of dietary carbohydrates [73]. We report, however, that primary lipogenic targets of MLXIPL signaling, such as key genes of *de novo* lipogenesis, show significant reduction in transcript expression in males. Taken together, these could indicate less efficient FA oxidation as well as the effects of dietary carbohydrates may be in the way of bean-induced protective health benefits for the liver in males.

### 4.7. Translational Considerations

In many respects, a vegan food pattern is a predominant type practiced globally. The significance of this is that it is well established that human health and well-being are maintained when all protein is obtained from plant sources. Moreover, globally, a substantial amount of plant protein is consumed from the botanical family Fabaceae. Within that family, the foods contributing to the protein are primarily either oilseed legumes (soy and peanut) or grain legumes, i.e., pulses (common bean, chickpea, lentil, and dry pea). Oilseed legumes predominate in economically developed countries, while pulses—in the remainder of the world. There, pulses are treated as a staple food crop, not a vegetable side dish. By definition, a staple food crop is eaten daily in large amounts as a primary source of energy and protein, and they are affordable and accessible. As such, pulses, with common bean as an example, could be leveraged to reduce the prevalence of fatty liver disease in developed countries featuring low levels of pulse consumption and in developing countries where the public health trends indicate that fatty liver disease is increasing in segments of the population with improving economic status preferring highly processed convenience foods. Collectively, this raises the important question: *is increased pulse consumption feasible given the amounts needed to secure disease preventive effects, e.g., on MAFLD?*

In our judgment, the answer is yes, but it will require increased prominence of stealth health approaches. Specifically, flours of each pulse can be prepared as minimally processed whole food ingredients and incorporated into foods for which consumers in various populations show a preference. As witnessed by the rapid increase in the release of pulse-containing food products, it is feasible. Beyond this, the effort will gain credibility in the biomedical community by identification of the specific gene/protein targets by which bean inhibits the development of a disease such as MAFLD and the specific chemical(s) that alters gene expression or protein activity. Whether the chemicals are an intrinsic component of bean and other pulses or are produced by interacting pulse food components with the gut microbiome and its associated immune component remains to be determined. Once that chemistry is understood, pulses can be processed to ensure that their use as food ingredients maintains the amount and form necessary to secure health benefits.

### 4.8. Limitations and Strengths

Our primary goal in the present study was to determine dose- and sex-dependent effects of bean consumption on liver protection from the obesogenic dietary environment and both quantitative chemical analyses and qualitative histopathology provide strong evidence of dose and biological sex dependent effects of bean consumption of dietary induced MAFLD. In addition, a secondary objective was to deduce potential molecular mechanisms underlying these observed phenotypes. Considering the multi-faceted nature of hepatic lipid metabolism, the results we report herein are only the tip of the iceberg and provide an initial overview of dietary bean effects on MAFLD prevention. Recognizing that transcriptional signatures are only one step in the regulatory cascade that determines protein activity and metabolite patterns, this work will serve to direct detailed analyses of effects of bean consumption on the phosphoproteome that are focused and guided by metabolomic analyses of plasma and liver. However, those analyses are considered beyond the scope of this investigation.

The translation of preclinical data to potential human relevance must always be done bearing in mind the limitations of each animal model. In the work reported herein, the model is well established for its value in studying metabolic dysfunction and the underlying findings have been demonstrated in two rodent species. This work was done in a preclinical model rather than in human subjects because of the paucity of information available about dose or the effect of biological sex. Additionally, a specific bean cultivar and a dose response design were used in male and female animals. Ultimately, mechanistic studies using genetically engineered mouse (GEM) methodology, e.g., knock-out strategies, will be required to establish causality. The work reported herein constitutes a foundation for in-depth, targeted analyses upon which genetic manipulations can be designed.

## 5. Conclusions

We demonstrate that consumption of common bean under isocaloric and obesogenic dietary conditions exerts potent protection from lipid accumulation in the liver in a dose- and sex-dependent manner. In particular:

❖ females exhibited lower hepatic lipid content at the 35% dose, whereas males achieved a similar reduction with the 70% dose of bean;
❖ common bean dose-responsively inhibited *de novo* lipogenesis in both sex cohorts;
❖ additionally, females showed greater capacity for FA oxidation, whereas males significantly reduced uptake of extracellular free FAs;
❖ bean consumption significantly affected genes involved in TAGs metabolism consistent with enhanced hydrolysis and diminished biosynthesis;
❖ bean-induced DEGs patterns indicated that reduced PPARα signaling and activated by dietary carbohydrates MLXIPL signaling may be at the core of lower susceptibility of male mice to dietary bean protection from hepatic steatosis;
❖ hepatic CYPs-regulated FA oxidation, oxidative stress, cholesterol metabolism, bile acid metabolism, and inflammation appear to also be significantly affected by increasing doses of beans in the diet and will be assessed in further communications.

Collectively, we provide strong evidence that incorporating common bean into the diet dose dependently protects both female and male mice from the diet-induced onset of MAFLD.

## Figures and Tables

**Table 1:** IPA analysis-ready identifiers mapped to DEGs within each comparison group pair.

Group Cohort	35% vs. 0% Bean	70% vs. 0% Bean	70% vs. 35% Bean
	*DEGs* ^1^	*DEGs* ^1^	*DEGs* ^1^
*Pre-filtered Mapped IDs* ^2^			
Females	3547	3579	3541
Males	3539	3539	3497
*Core Analysis-ready IDs* ^3^			
Females	461	966	389
Males	394	1130	535
***TOTAL DEGs*** ^4^	*66*,*624*		

^1^ Differentially expressed genes; ^2^ All mapped to DEGs and duplicate-resolved identifiers; ^3^ Filtered by *p*-value < 0.05. ^4^ Total amount of observed DEGs from CLC Genomics Workbench output dataset.

## Data Availability

The RNA sequencing data reported herein is being submitted to the Gene Expression Omnibus, a database for gene expression profiling managed by the National Center for Biotechnology Information. Please contact the corresponding author for the GEO accession number.

## Supplementary Materials

The following supporting information can be downloaded at: https://www.mdpi.com/article/10.3390/nu15030526/s1, Table S1: Diet formulations; Table S2: IPA Input; Table S3: Toxicity functions; Table S4: DEGs; Table S5: Canonical Pathways; Table S6: Upstream regulators; Figure S1: Effect of dietary bean on the liver lipid content; Figure S2: Dietary bean effects on expression of hepatic fatty acid transport genes in female and male mice; Figure S3: Expression of genes involved in *de novo* lipogenesis in the livers of female and male mice upon consumption of beans; Figure S4: Dietary bean dose-dependently affects expression of acyl-CoA synthetases in the livers of female and male mice; Figure S5: Consumption of beans promotes sex-dependent changes in gene expression regulating mitochondrial β-oxidation in the livers of mice; Figure S6: Effects of dietary bean on peroxisomal β-oxidation in the livers of mice; Figure S7: Dietary beans affect expression of genes involved in lipid droplet and lipoprotein formation and clearance in both female and male mice; Figure S8: Effects of dietary bean on gene expression involved in triacylglycerol metabolism in both female and male mice; Figure S9: Observed DEGs among the predicted significant upstream regulators.
