# Supplementary material for: Thwarting Metabolic Dysfunction-Associated Fatty Liver Disease (MAFLD) with Common Bean: Dose- and Sex-Dependent Protection against Hepatic Steatosis
Source: Nutrients. 2023 Jan 19;15(3):526. doi: 10.3390/nu15030526 (PMC9920904; doi:10.3390/nu15030526)
Supplement: Supplementary file 1 [file nutrients-15-00526-s001.zip › Supplemental Figures. Thwarting metabolic dysfunction-associated fatty liver disease (MAFLD) with dry beans.pdf]

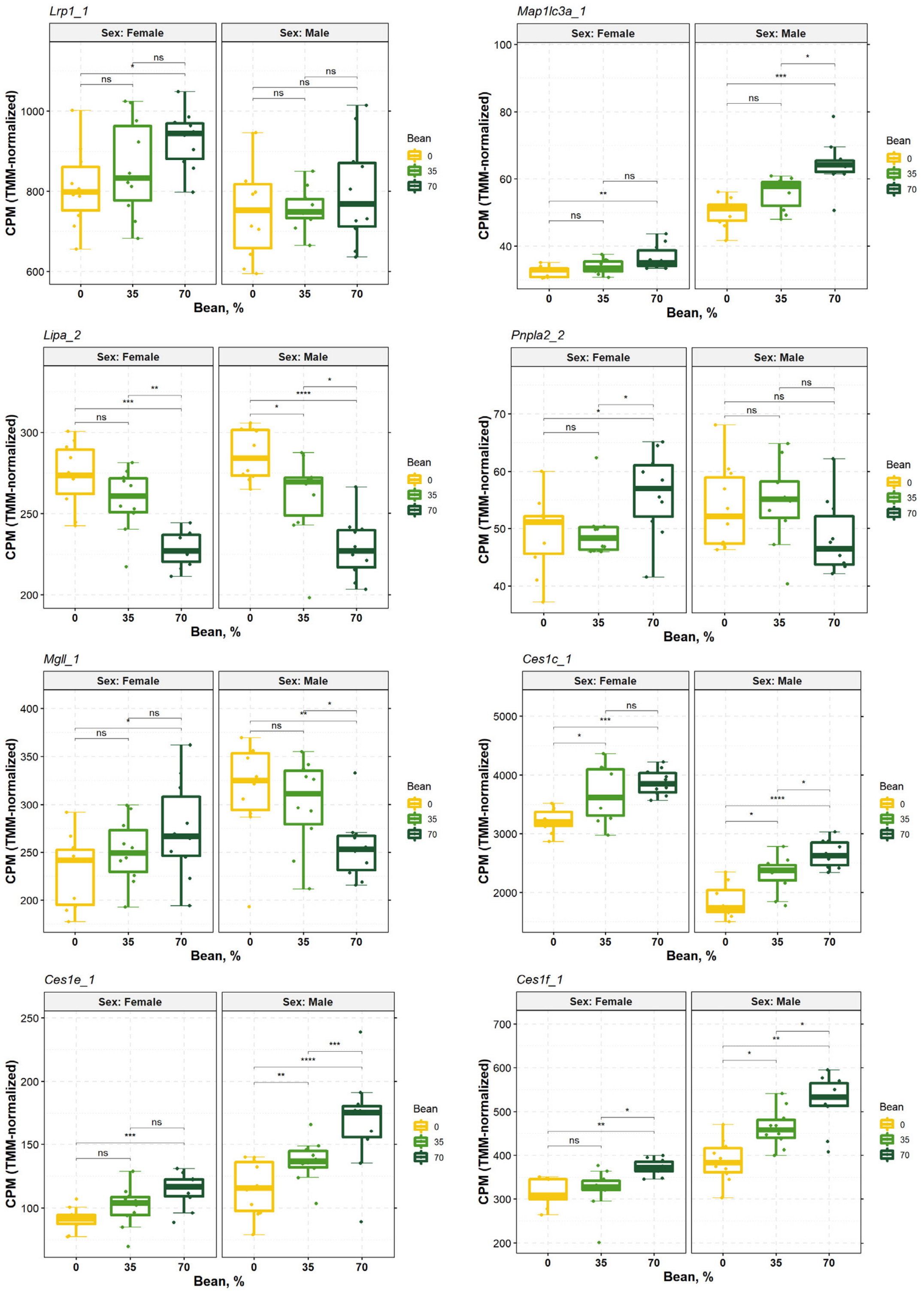

Lrp1_1
Map1lc3a_1
Lipa_2
Pnpla2_2
Mgll_1
Ces1c_1
Ces1e_1
Ces1f_1
Sex: Female
Sex: Male
CPM (TMM-normalized)
Bean, %
Bean
0
35
70

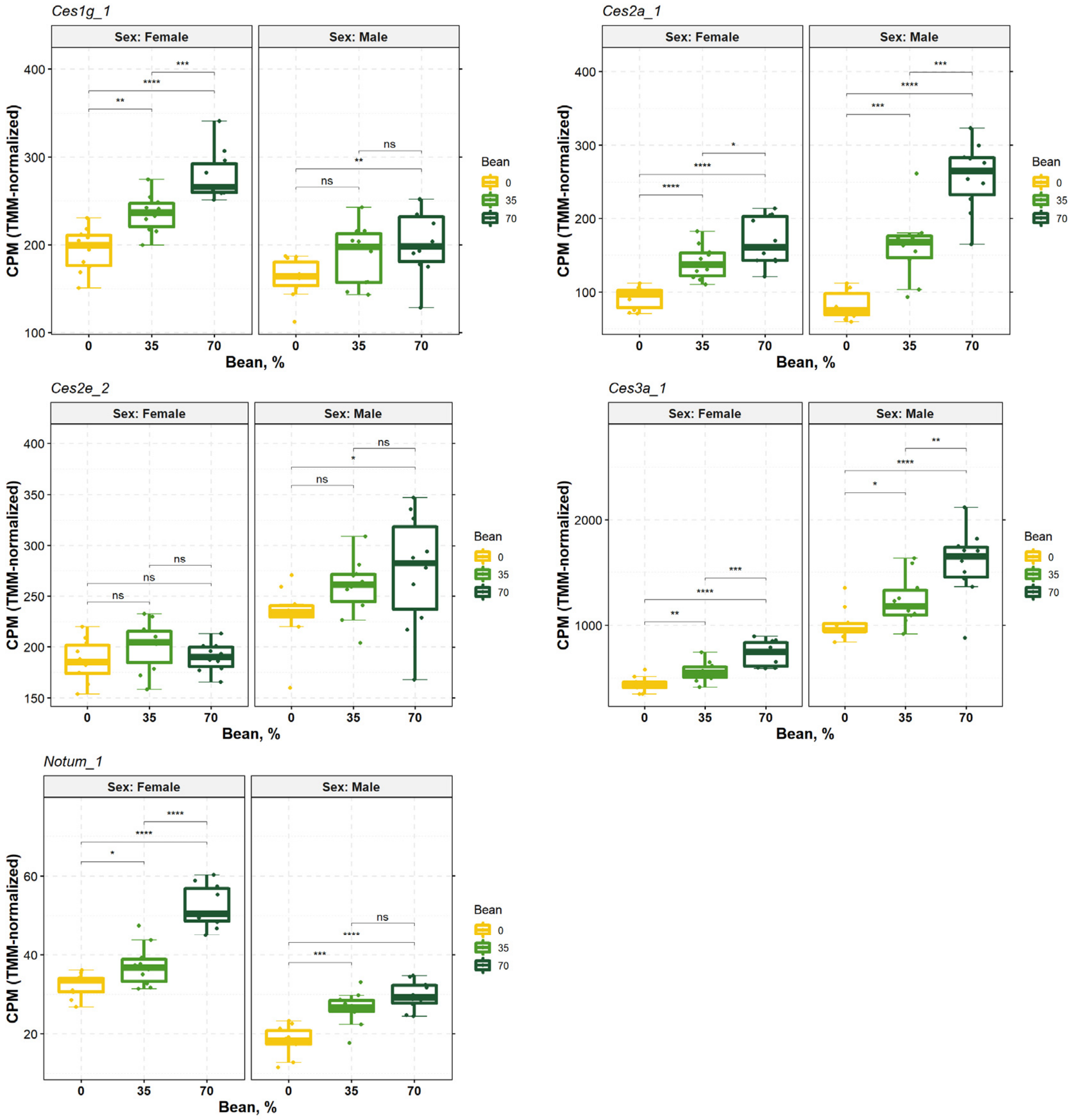

Figure S1. Effects of dietary bean on gene expression involved in triacylglycerol metabolism in both female and male mice. Differential expression analysis performed in CLC Genomic Workbench. * p-value < 0.05; ** p-value < 0.01; *** p-value < 0.001; **** p-value < 0.0001

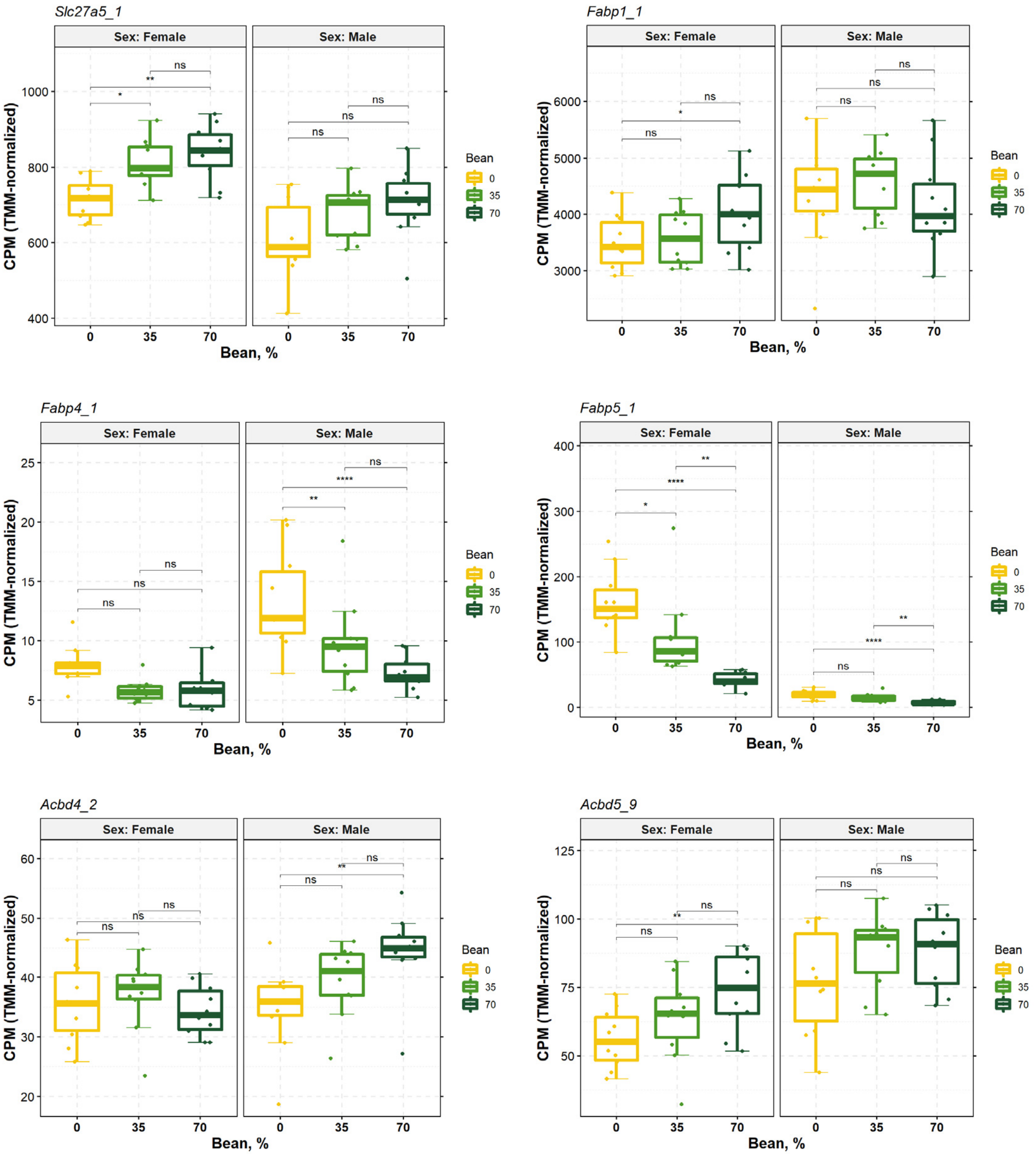

Figure S2. Dietary bean effects on expression of hepatic fatty acid transport genes in female and male mice. Differential expression analysis performed in CLC Genomic Workbench. * p-value < 0.05; ** p-value < 0.01; *** p-value < 0.001; **** p-value < 0.0001

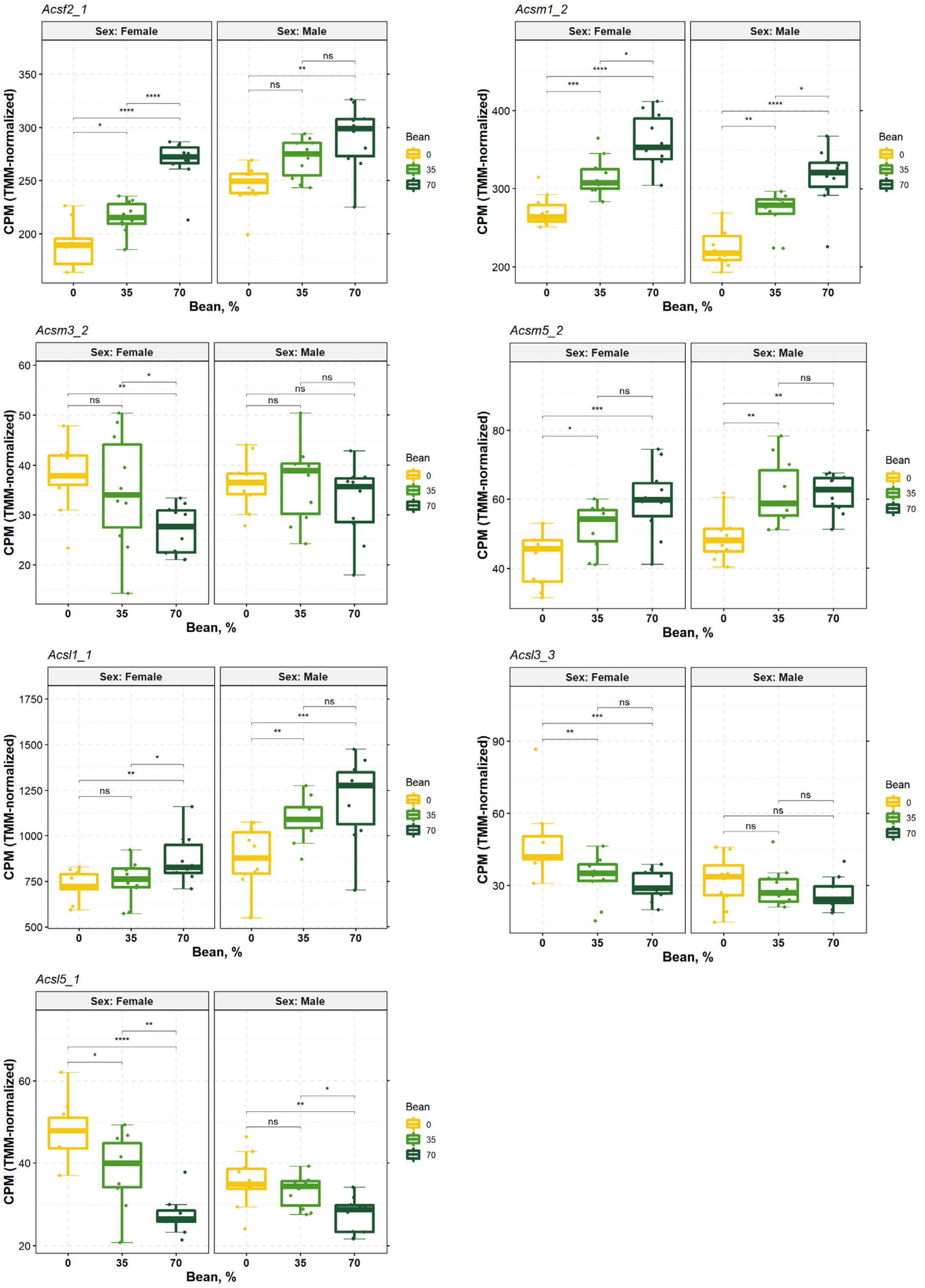

Figure S3. Dietary bean dose-dependently affects expression of acyl-CoA synthetases in the livers of female and male mice. Differential expression analysis performed in CLC Genomic Workbench. * p-value < 0.05; ** p-value < 0.01; *** p-value < 0.001; **** p-value < 0.0001

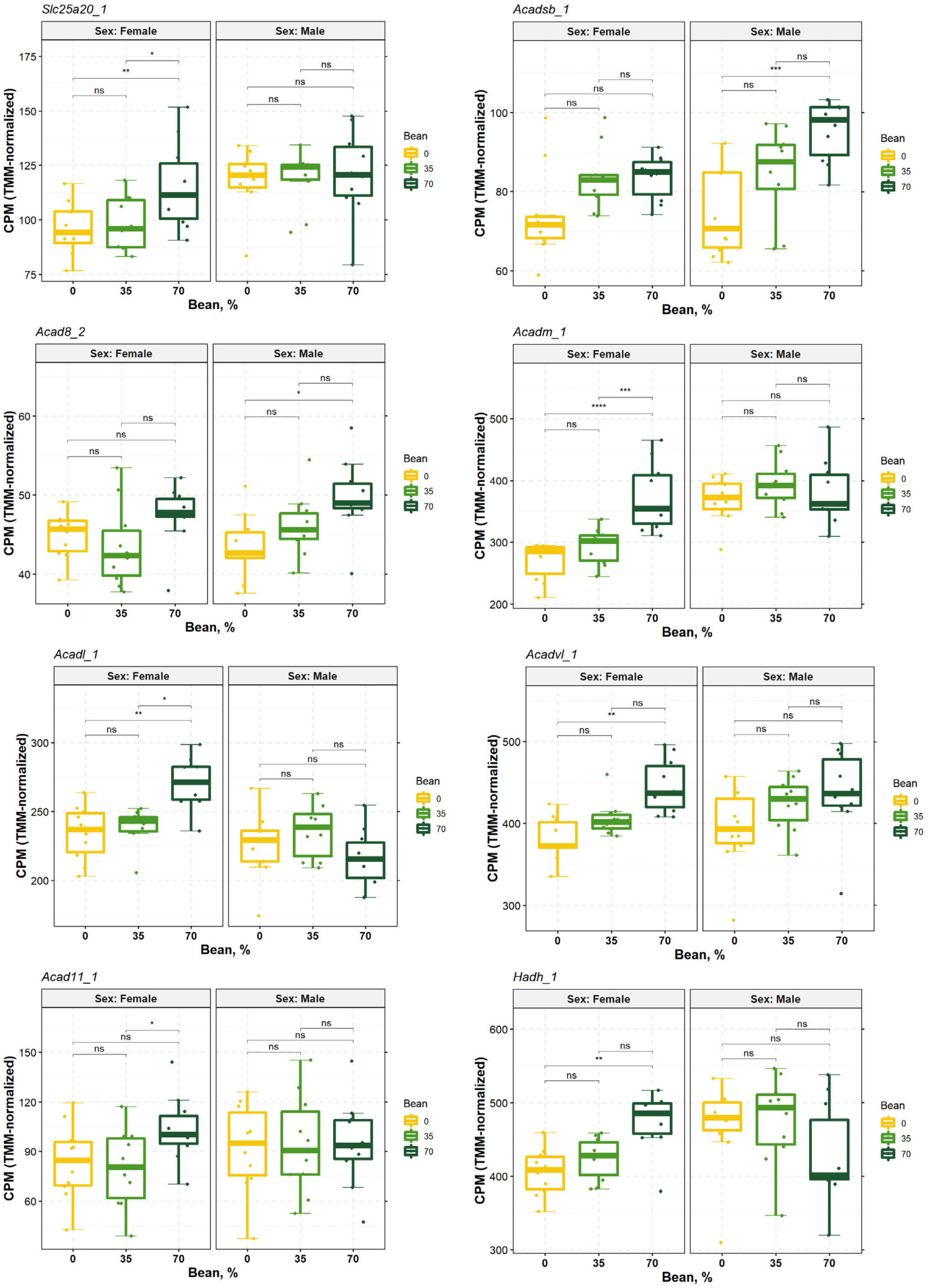
Slc25a20_1
Acadsb_1
Acad8_2
Acadm_1
Acadl_1
Acadvl_1
Acad11_1
Hadh_1
Sex: Female
Sex: Male
CPM (TMM-normalized)
Bean, %
Bean
0
35
70
ns
*
**
***
****

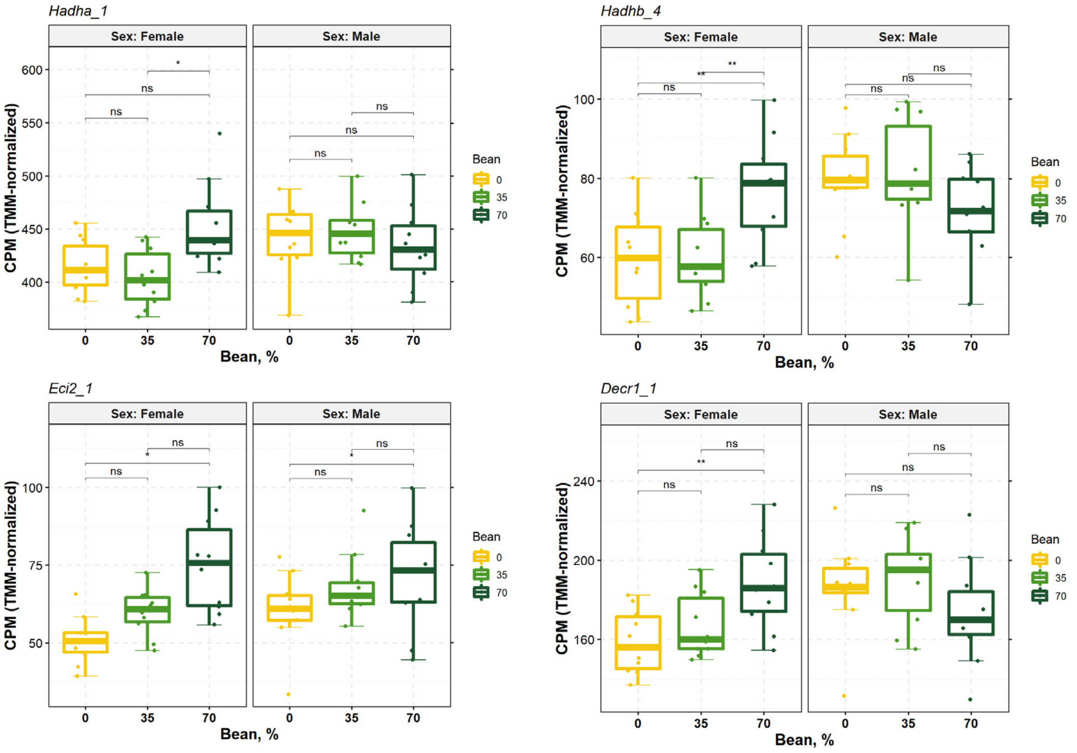

Figure S4. Consumption of beans promotes sex-dependent changes in gene expression regulating mitochondrial β-oxidation in the livers of mice. Differential expression analysis performed in CLC Genomic Workbench. * p-value < 0.05; ** p-value < 0.01; *** p-value < 0.001; **** p-value < 0.0001

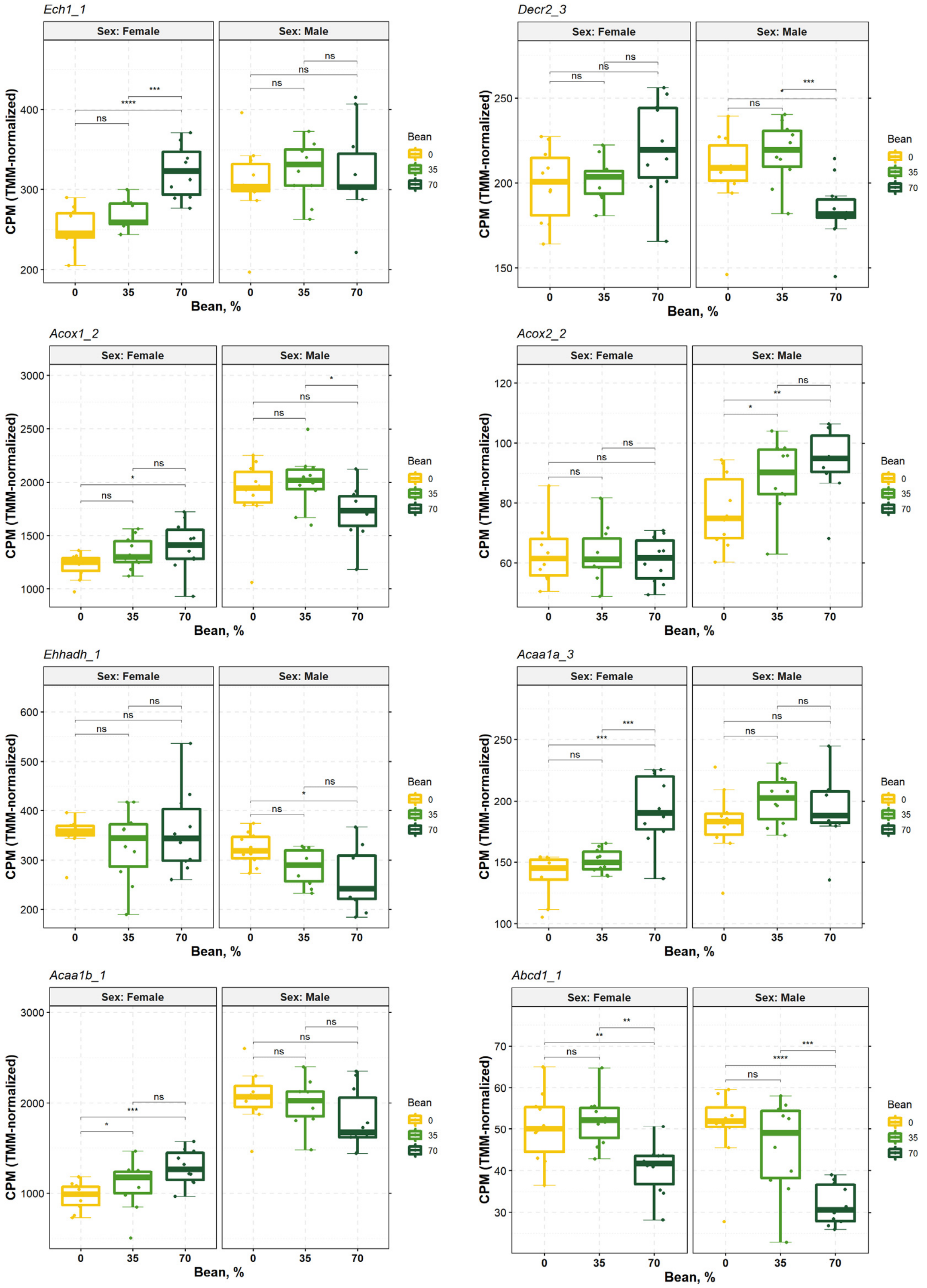

Figure S5. Effects of dietary bean on peroxisomal β-oxidation in the livers of mice. Differential expression analysis performed in CLC Genomic Workbench. * p-value < 0.05; ** p-value < 0.01; *** p-value < 0.001; **** p-value < 0.0001

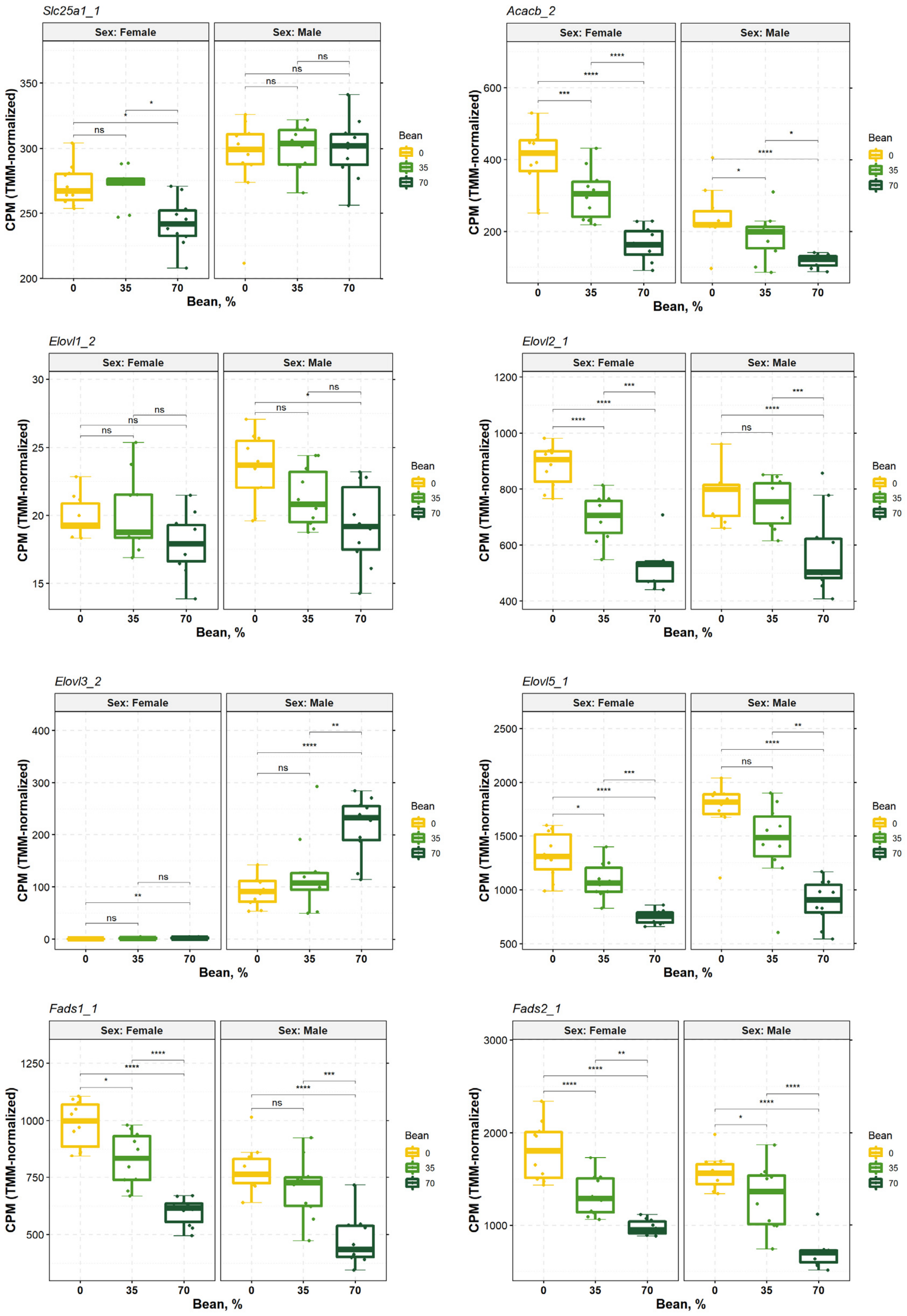

Figure S6. Expression of genes involved in *de novo* lipogenesis in the livers of female and male mice upon consumption of beans. Differential expression analysis performed in CLC Genomic Workbench. * p-value < 0.05; ** p-value < 0.01; *** p-value < 0.001; **** p-value < 0.0001

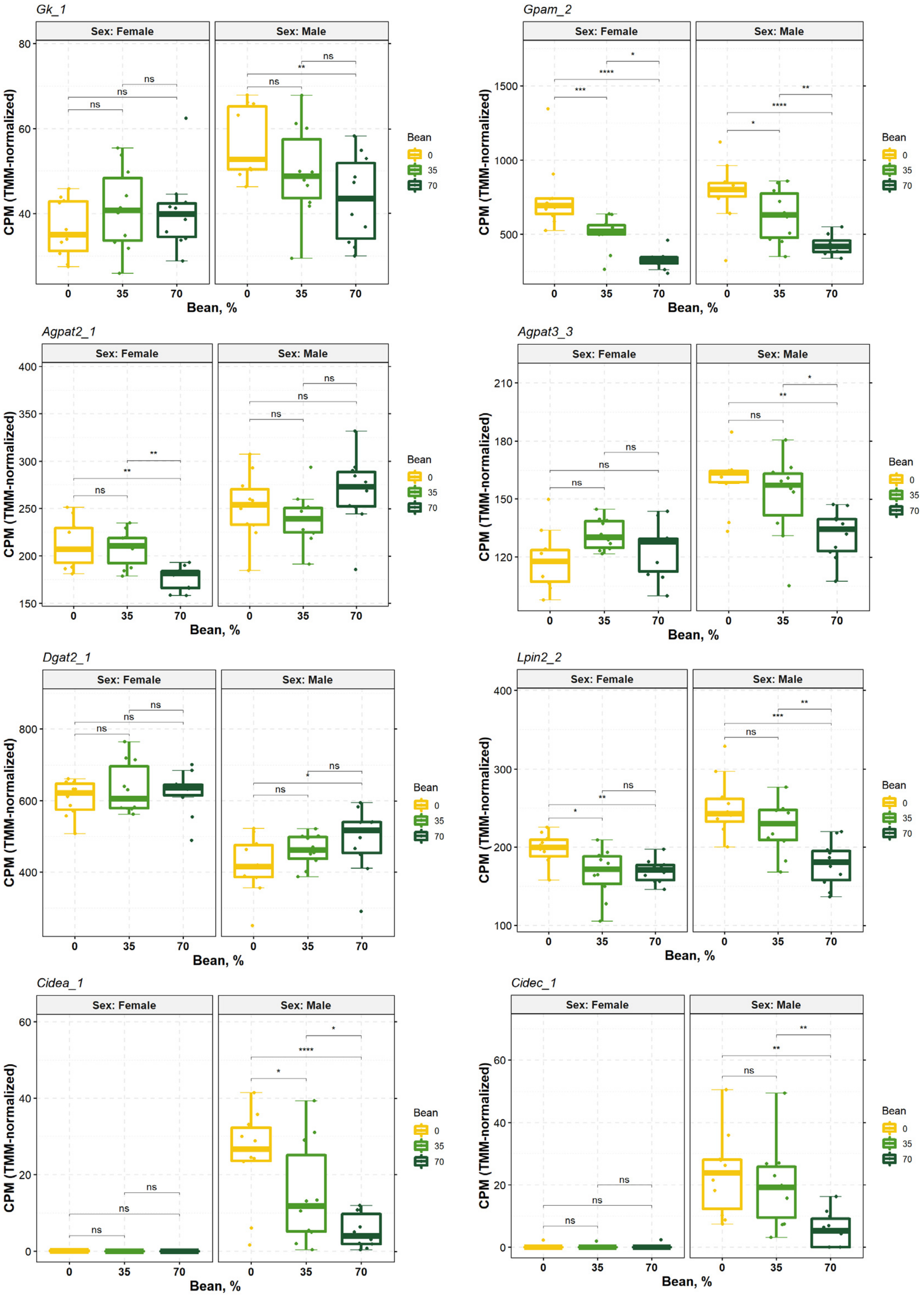

Gk_1
Sex: Female
Sex: Male
CPM (TMM-normalized)
80
60
40
ns
ns
ns
ns
**
ns
0
35
70
Bean, %
Bean
0
35
70
Gpam_2
1500
1000
500
***
****
*
*
****
**
Agpat2_1
400
350
300
250
200
150
ns
**
**
ns
ns
ns
Agpat3_3
210
180
150
120
ns
ns
ns
ns
**
*
Dgat2_1
800
600
400
ns
ns
ns
ns
*
ns
Lpin2_2
400
300
200
100
*
**
ns
ns
***
**
Cidea_1
60
40
20
0
ns
ns
ns
*
****
*
Cidec_1
60
40
20
0
ns
ns
ns
ns
**
**

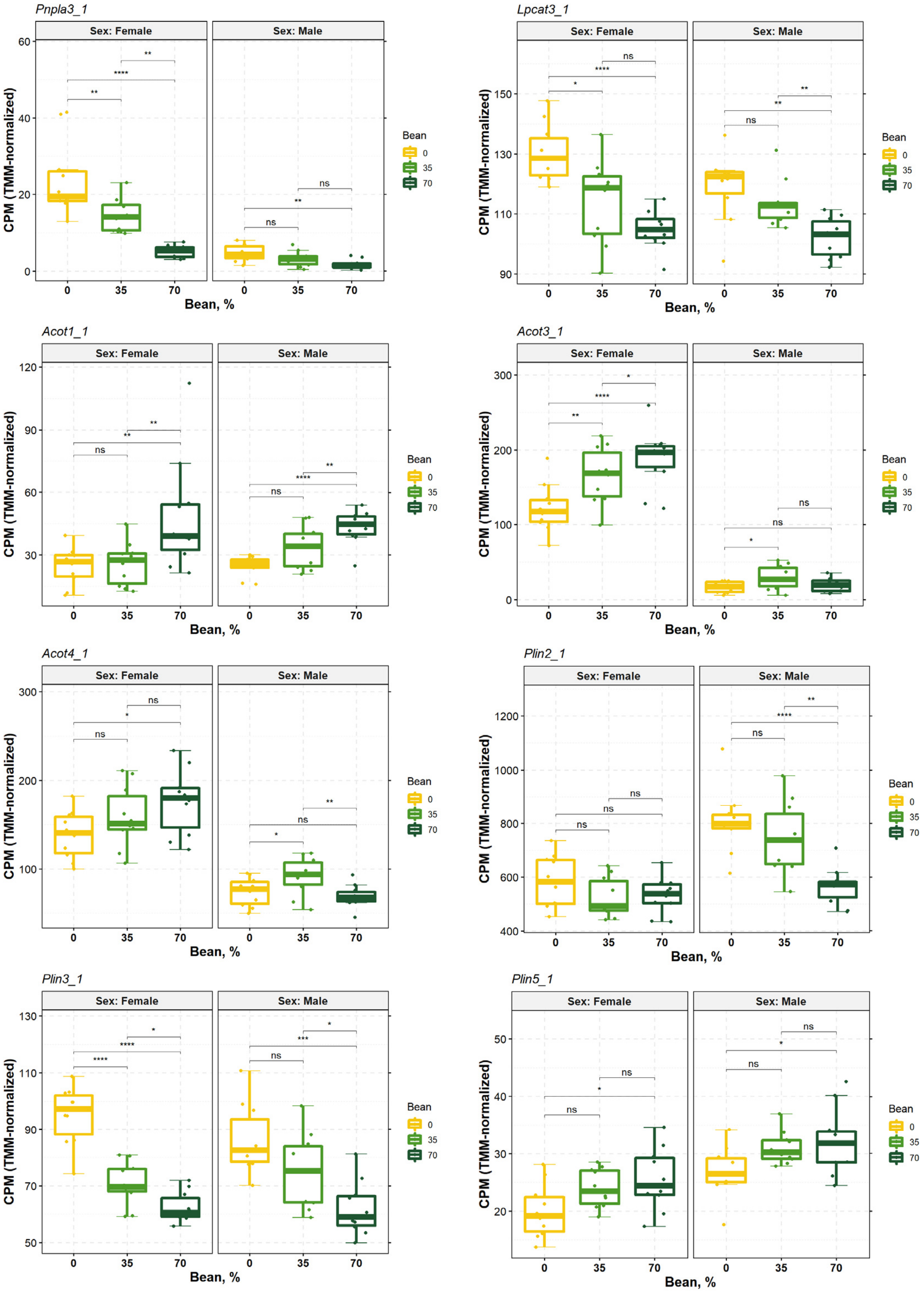
Pnpla3_1
Lpcat3_1
Acot1_1
Acot3_1
Acot4_1
Plin2_1
Plin3_1
Plin5_1
Sex: Female
Sex: Male
CPM (TMM-normalized)
Bean, %
Bean
0
35
70

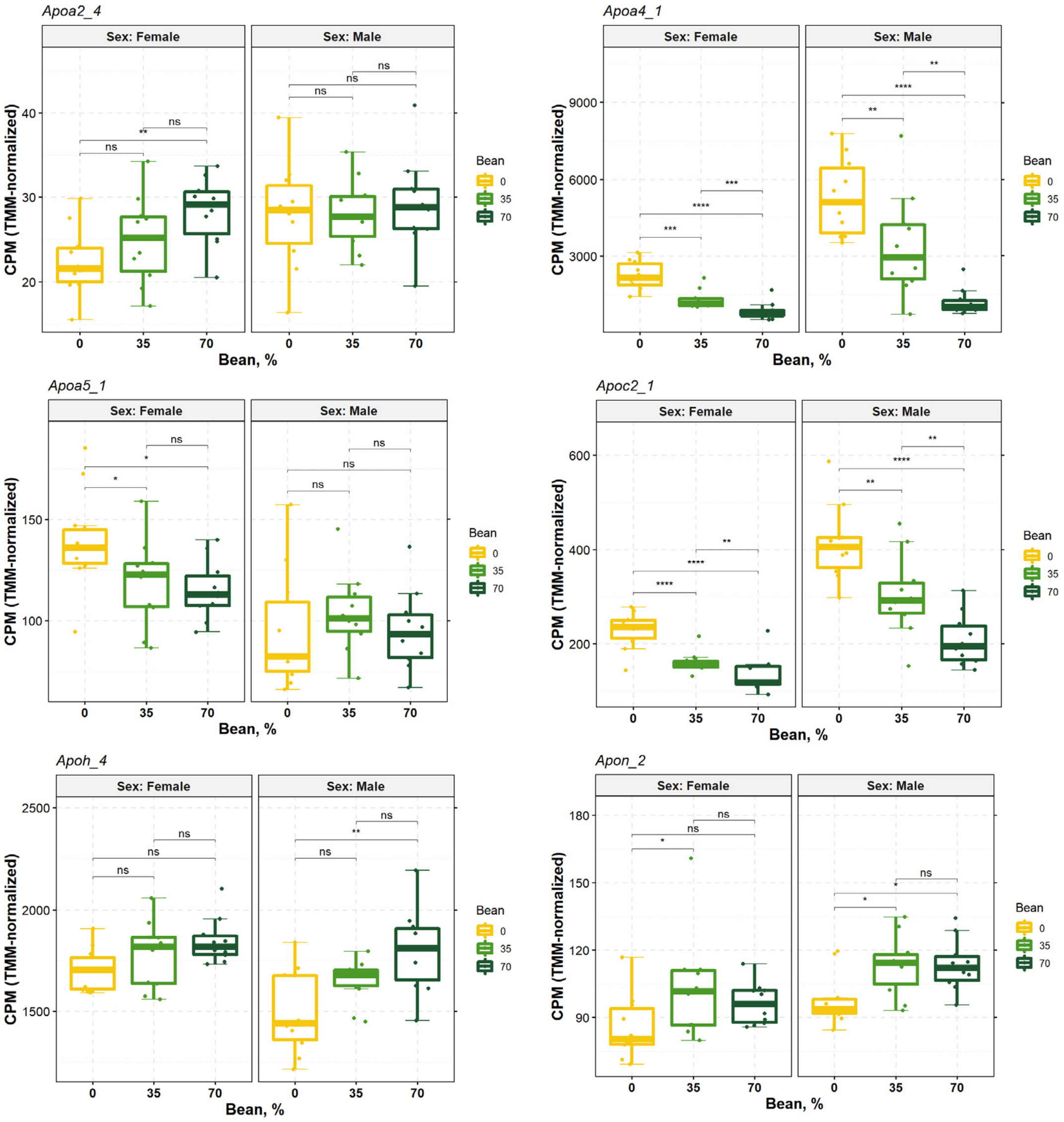

Figure S7. Dietary beans affect expression of genes involved in lipid droplet and lipoprotein formation and clearance in both female and male mice. Differential expression analysis performed in CLC Genomic Workbench. * p-value < 0.05; ** p-value < 0.01; *** p-value < 0.001; **** p-value < 0.0001

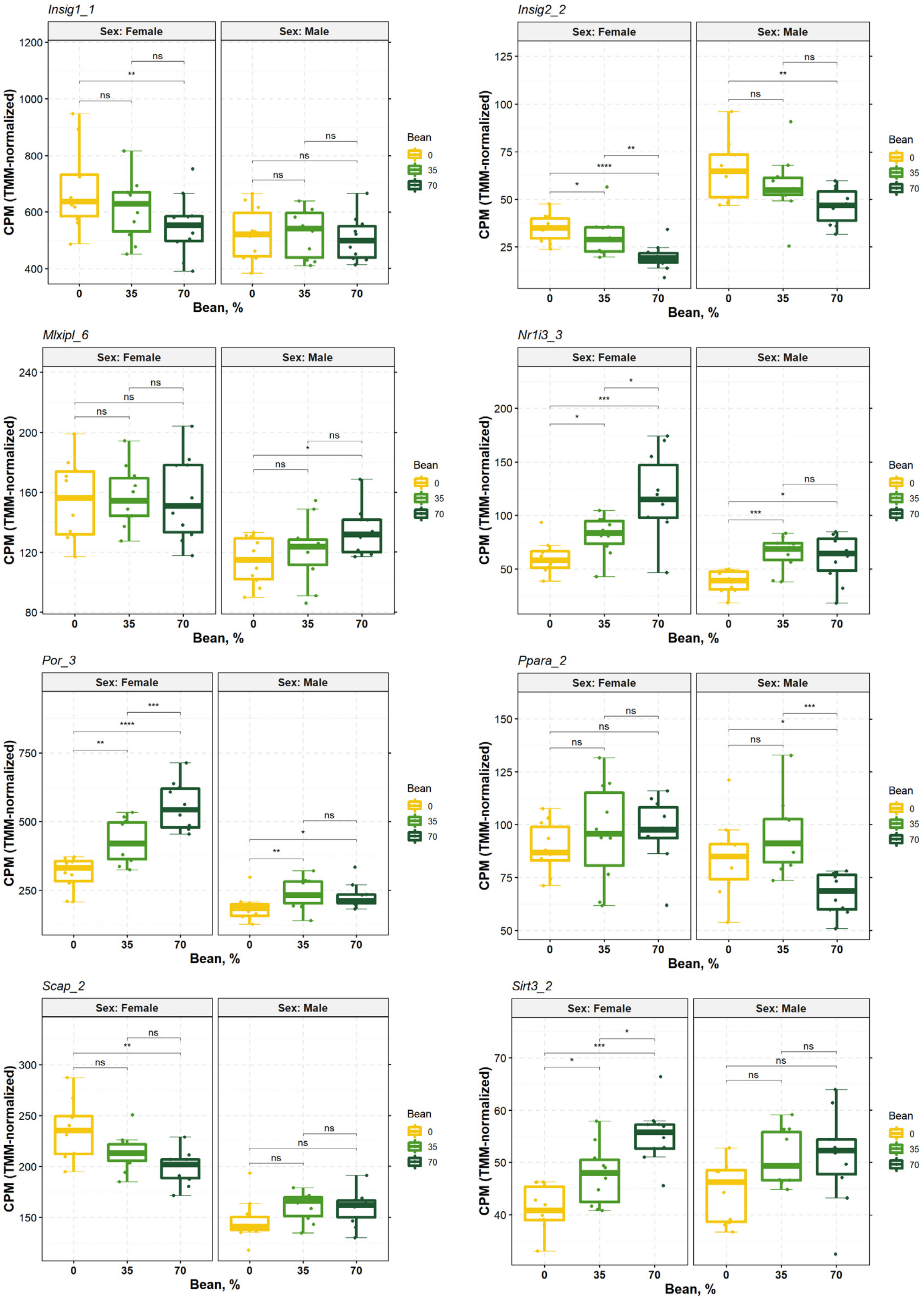
Insig1_1
Insig2_2
Mlxipl_6
Nr1i3_3
Por_3
Ppara_2
Scap_2
Sirt3_2
Sex: Female
Sex: Male
CPM (TMM-normalized)
Bean, %
Bean
0
35
70
ns
*
**
***
****

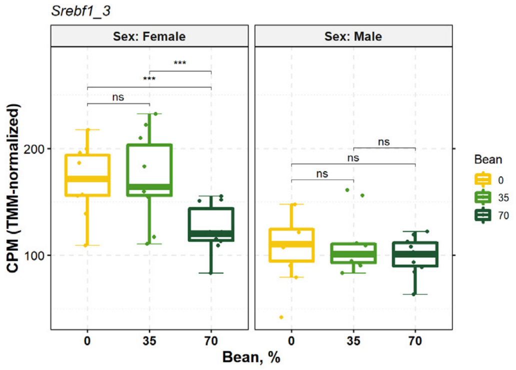

Figure S8. Observed DEGs among the predicted significant upstream regulators. Differential expression analysis performed in CLC Genomic Workbench. * p-value < 0.05; ** p-value < 0.01; *** p-value < 0.001; **** p-value < 0.0001
